# PRRSV GP5 inhibits the antivirus effects of chaperone-mediated autophagy by targeting LAMP2A

**DOI:** 10.1128/mbio.00532-24

**Published:** 2024-06-28

**Authors:** Wen Li, Mengting Zhang, Yueshuai Wang, Shijie Zhao, Pengli Xu, Zhiying Cui, Jing Chen, Pingan Xia, Yina Zhang

**Affiliations:** 1 College of Veterinary Medicine, Henan Agricultural University, Zhengzhou, Henan, China; 2 College of Life Science, Henan Agricultural University, Zhengzhou, Henan, China; 3 Ministry of Education Key Laboratory for Animal Pathogens and Biosafety, Zhengzhou, Henan, China; The Ohio State University School of Medicine, Columbus, Ohio, USA; Instituto Nacional de Investigacion y Tecnologia Agraria y Alimentaria, Madrid, Spain

**Keywords:** PRRSV, GP5, CMA, LAMP2A, GFAP

## Abstract

Autophagy is an important biological process in host defense against viral infection. However, many viruses have evolved various strategies to disrupt the host antiviral system. Porcine reproductive and respiratory syndrome virus (PRRSV) is a typical immunosuppressive virus with a large economic impact on the swine industry. At present, studies on the escape mechanism of PRRSV in the autophagy process, especially through chaperone-mediated autophagy (CMA), are limited. This study confirmed that PRRSV glycoprotein 5 (GP5) could disrupt the formation of the GFAP-LAMP2A complex by inhibiting the MTORC2/PHLPP1/GFAP pathway, promoting the dissociation of the pGFAP-EF1α complex, and blocking the K63-linked polyubiquitination of LAMP2A to inhibit the activity of CMA. Further research demonstrated that CMA plays an anti-PRRSV role by antagonizing nonstructural protein 11 (NSP11)-mediated inhibition of type I interferon (IFN-I) signaling. Taken together, these results indicate that PRRSV GP5 inhibits the antiviral effect of CMA by targeting LAMP2A. This research provides new insight into the escape mechanism of immunosuppressive viruses in CMA.

**IMPORTANCE:**

Viruses have evolved sophisticated mechanisms to manipulate autophagy to evade degradation and immune responses. Porcine reproductive and respiratory syndrome virus (PRRSV) is a typical immunosuppressive virus that causes enormous economic losses in the swine industry. However, the mechanism by which PRRSV manipulates autophagy to defend against host antiviral effects remains unclear. In this study, we found that PRRSV GP5 interacts with LAMP2A and disrupts the formation of the GFAP-LAMP2A complex, thus inhibiting the activity of CMA and subsequently enhancing the inhibitory effect of the NSP11-mediated IFN-I signaling pathway, ultimately facilitating PRRSV replication. Our study revealed a novel mechanism by which PRRSV escapes host antiviral effects through CMA, providing a potential host target, LAMP2A, for developing antiviral drugs and contributing to understanding the escape mechanism of immunosuppressive viruses.

## INTRODUCTION

Porcine reproductive and respiratory syndrome (PRRS), caused by the porcine reproductive and respiratory syndrome virus (PRRSV), is one of the most important infectious viral diseases affecting the pig industry worldwide (1). PRRSV is an enveloped single-stranded positive-sense RNA virus in the *Arteriviridae* family*,* and the genome is approximately 15 kb in length, with at least 10 known open reading frames (ORFs). The majority of the genome (ORF1a and ORF1b) encodes nonstructural proteins (NSPs) essential for viral replication (NSP1α, NSP1β, NSP2-6, NSP7α, NSP7β, and NSP8-12), whereas the remaining part (ORFs 2–7) encodes structural proteins (GP2a, GP2b, GP3-GP5, GP5a, M, and N) (2). GP5, which has a neutralizing epitope, is the major virulence determinant among structural proteins and plays an important role in the pathogenicity and immune evasion of PRRSV (3–6).

Autophagy is a vital process for maintaining cellular homeostasis, particularly the balance between protein synthesis and degradation (7). In mammalian cells, cellular cargo is delivered to the lysosome for degradation or recycling. According to the mode of cargo delivery, autophagy can be divided into macroautophagy, microautophagy, and chaperone-mediated autophagy (CMA) (8–11). CMA is a selective type of autophagy, and its process can be roughly divided into four steps. In the initial phase of CMA, substrate proteins containing a KFERQ-like pentapeptide motif (named the CMA motif) are recognized by heat shock cognate protein 70 (HSC70) (step 1). Then, the proteins are translocated to the surface of the lysosomal membrane and bind to the cytosolic tail of lysosome-associated membrane protein type 2A (LAMP2A) (12–14) (step 2). Substrate binding induces the organization of this single-spanning membrane protein into a multimeric complex (translocation complex) that facilitates the translocation process (step 3). Once the substrate protein crosses the lysosomal membrane, the translocation complex disassembles into monomers of LAMP2A, enabling a new cycle of substrate binding and translocation (15) (step 4). The disassembly of LAMP2A occurs in the lipid microdomains of lysosomal membranes, wherein LAMP2A undergoes cleavage by cathepsin A and metalloproteinases (16, 17).

In addition to HSC70 and LAMP2A, the lysosome has regulators that directly modulate CMA activity. For instance, a pair of regulators, glial fibrillary acidic protein (GFAP)-elongation factor 1α (EF1α), modulate the assembly-disassembly dynamics of LAMP2A in a GTP-dependent manner (18). There are two forms of GFAP in the lysosomal membrane: unmodified GFAP and phosphorylated GFAP (pGFAP). Unmodified GFAP binds to LAMP2A in the multimeric complex and contributes stabilization. pGFAP has a low binding affinity for LAMP2A and is associated with EF1α in the form of the pGFAP-EF1α complex. After substrate translocation, EF1α is released from the lysosomal membrane, allowing pGFAP to access unmodified GFAP. GFAP has a greater affinity to form a dimer with pGFAP than to bind to LAMP2A, which results in the formation of a GFAP-pGFAP dimer and the disassembly of LAMP2A as unmodified GFAP leaves the translocation complex (18). A second mechanism that regulates the dynamics of LAMP2A assembly-disassembly into the translocation complex involves a CMA regulatory axis. It comprises the mammalian target of rapamycin complex 2 (MTORC2, a CMA inhibitor), the pleckstrin homology domain, and leucine-rich repeat protein phosphatase 1 (PHLPP1, a CMA activator), and their common downstream target the AKT serine/threonine kinase (AKT, a CMA inhibitor) (19). Phosphorylation of the serine/threonine kinase AKT by MTORC2 increases the phosphorylation of GFAP (19). Continuous phosphorylation of GFAP drives unmodified GFAP away from multimerized LAMP2A, leading to subsequent destabilization of this translocation complex and reduced CMA activity. PHLPP1 is recruited to the lysosomal membrane in a Rac1-dependent manner, and it enhances CMA through the dephosphorylation of lysosomal AKT, thus stabilizing the translocation complex (19). Therefore, the levels of CMA effectors and modulators should be analyzed to yield useful insights into CMA activity in cells.

Autophagy and viral infection are closely interconnected. Although autophagy promotes the clearance of cytoplasmic material, some viruses adopt various strategies to manipulate multiple steps during autophagy to meet the final goal of survival and propagation (20). Some viruses exploit incomplete autophagic flux for their replication. Hou et al. reported that the ORF7a protein of SARS-CoV-2 initiates autophagy and limits autophagosome-lysosome fusion to promote virus replication (21). Similarly, the P protein of human parainfluenza virus type 3 (HPIV3) can block autophagosome-lysosome fusion to increase virus production (22). Zika virus (ZIKV) promotes the degradation of karyopherin subunit alpha 2 (KPNA2) through CMA induction to maintain its replication (23).

At present, studies on autophagy induced by PRRSV infection have focused mainly on the mechanism by which PRRSV or its NSPs underlie autophagosome formation. Sun et al. showed that PRRSV infection induced incomplete autophagic flux (24). Zhang et al. revealed that PRRSV NSP3 and NSP5 induced the formation of autophagosomes (25). Zhou et al. discovered that PRRSV NSP5 impairs the fusion of autophagosomes with lysosomes, thereby blocking autophagic flux (26). However, the regulatory mechanism of PRRSV in the autophagy process is quite limited. This study is the first to confirm that PRRSV GP5 could hijack LAMP2A to inhibit the antiviral effects of CMA. This finding is expected to provide new insight into the viral evasion mechanism.

## MATERIALS AND METHODS

### Cells and virus

HEK293T (ATCC CRL-11268) and MARC-145 cells (CCLV-RIE 0277) were cultured in Dulbecco’s modified Eagle’s medium (DMEM; Solarbio Life Sciences, 12110) supplemented with 10% fetal bovine serum (FBS) (ExCell Bio, FSP500). 3D4/21 (ATCC CRL-2843) and primary porcine alveolar macrophages (PAMs; obtained from the bronchoalveolar lavage of a 4-week-old-specific pathogen-free swine) were cultured in RPMI Medium 1640 (Solarbio Life Sciences, 31800) supplemented with 10% FBS. PRRSV strain Hn07-1 (KX766378) was generated in MARC-145 cells. Sendai virus (SeV) was preserved in our laboratory.

### Reagents

Anti-LC3B (2775), anti-SQSTM1 (8025), anti-phospho-AKT1 (Ser473; 4060), anti-HSC70 (8444), anti-MTOR (2972), anti-pMTOR (S2448; 5536), and anti-Flag rabbit monoclonal antibodies (mAbs; 14793) were purchased from Cell Signaling Technology. Anti-Myc rabbit polyclonal antibodies (pAb; R1208-1), anti-GST mouse mAb (EM80701), anti-beta Actin (ET1702-67) and anti-LAMP2A (ET1601-24) rabbit mAbs, Alexa Fluor 647 goat anti-mouse IgG antibody (HA1127), and Alexa Fluor 647 goat anti-rabbit IgG antibody (HA1106) were purchased from Hangzhou HuaAn Biotechnology. Anti-PHLPP1(abs118646), anti-GFAP (abs115050), and anti-phospho-GFAP (Ser8; abs155727) rabbit pAbs were purchased from Absin Biotech Co., Ltd. Anti-HA mouse pAb (66006-2-Ig) was purchased from Proteintech Group, Inc. Mouse anti-PRRSV-N and mouse anti-PRRSV-GP5 mAbs were produced by our laboratory. Fluorescein isothiocyanate (FITC)-labeled goat anti-mouse IgG antibody (172-1806), FITC-labeled goat anti-rabbit IgG antibody (172-1506), horseradish peroxidase (HRP)-labeled goat anti-mouse IgG antibody (074-1806), and HRP-labeled goat anti-rabbit IgG antibody (074-1506) were purchased from Kirkegaard & Perry Laboratories, Inc. Dylight 405 goat anti-rabbit IgG antibody (A23120) was obtained from Abbkine Scientific Co., Ltd. Alexa Fluor 546 donkey anti-rabbit IgG antibody (A11040), Alexa Fluor 546 donkey anti-mouse IgG antibody (A11030), Lipofectamine 2000 (11668019), and PageRuler Prestained Protein Ladder (26617) were purchased from Thermo Fisher Scientific Co., Ltd. Anti-Flag M2 mouse mAb (F1804), anti-Flag M2 affinity gel (A2220), and dimethyl sulfoxide (DMSO; 472301) were purchased from Sigma‒Aldrich. Leupeptin (HY-18234A), cycloheximide (CHX; HY-12320), and 3-MA (HY19312) were purchased from MedChem Express. MG132 (S1748), NP-40 Lysis Buffer (P0013F), phenylmethanesulfonyl fluoride (PMSF; ST506), Protein A/G Agarose (P2005), and Dual Luciferase Reporter Gene Assay Kit (RG028) were purchased from Beyotime Biotechnology. Rapamycin (R8140), chloroquine (CQ; IC4440), 4′,6-diamidino-2-phenylindole (DAPI; C0065), and Antifade Mounting Medium (S2100) were purchased from Solarbio Life Sciences. TRIzol reagent (R401-01), HiScript II 1st Strand cDNA Synthesis Kit (R211-01), Phanta Max Super-Fidelity DNA Polymerase (P505-d1), Rapid Taq Master Mix (P222-01), and ChamQ Universal SYBR qPCR Master Mix (Q711-02) were purchased from Vazyme Biotech Co., Ltd.

### Plasmid construction

Endogenous microtubule-associated protein 1 light chain 3 (LC3), which was cloned from the PAM genome (NM_001170827), was ligated into the pEGFP-C3 vector (Clontech, 6082-1). GP3, GP4, GP5, M, and N, which were cloned from the PRRSV Hn07-1 genome (KX766378), were inserted into the pCMV-Flag-N vector (Clontech, 635688). GP5 mutants, such as GP5ΔCMA, GP5 (QK-AA), GP5Δ33-65, GP5Δ66-88, GP5Δ89-102, GP5Δ103-125, GP5Δ126-201, GP5 (66-88), and GP5 (126-201), were amplified by overlapping PCR from pCMV-Flag-GP5. HSC70, LAMP2A, GFAP and EF1α, which were cloned from the PAM genome (NM_001243907, NM_001244255, XM_005668709, and DQ673096), were ligated into pCMV-Flag-N, pCMV-Myc-N (Clontech, 631604), pEGFP-C3 (Clontech, 6082-1), and/or pmCherry-C1 (Clontech, 632524). LAMP2A mutants, such as LAMP2AΔSignal, LAMP2AΔLumenal 1, LAMP2AΔHinge, LAMP2AΔLumenal 2, LAMP2AΔTM, and LAMP2AΔTail, were amplified by overlapping PCR from pCMV-Flag-LAMP2A. GFAP mutants, such as GFAP (S8D) and GFAP (S8A), were, respectively, amplified with overlapping PCR from both GFP-GFAP and Flag-GFAP. All plasmid constructs were confirmed by sequencing. The sequences of primers used for plasmid construction are listed in Table S1. pIFN-β-luc, pRL-TK, pCMV-Flag-NSP4, pCMV-Flag-NSP5, pCMV-Flag-NSP7, pCMV-Flag-NSP9, pCMV-Flag-NSP10, pCMV-Flag-NSP11, and pCMV-Flag-NSP12 were preserved in our laboratory.

### RNA quantification

Total RNA was isolated according to the manufacturer’s instructions for the TRIzol reagent kit, and the isolated RNA was reverse transcribed into cDNA using a reverse transcription kit. Real-time quantitative PCR (RT-qPCR) was then performed using the CFX 96 Touch System (Bio-Rad, Hercules, CA, USA) in a 96-well plate. β-Actin was used as an internal control for normalization. The results were calculated using the 2^−ΔΔCt^ method. The sequences of the primers used for RT‒qPCR are listed in Table S1.

### Cell transfection and viral infection

For cell transfection, HEK293T, MARC-145, or 3D4/21 cells were seeded in designated plates or glass coverslips at a suitable seeding density according to the experimental scheme. When the cells reached 70%–80% confluence, the plasmids were transfected with Lipofectamine 2000 Transfection Reagent according to the manufacturer’s instructions. Finally, they were subjected to western blotting analysis to detect the protein levels. For cell infection, MARC-145 cells or PAMs were infected with PRRSV at an MOI of 1 for the indicated times, and the lysates from these cells were then analyzed using western blotting assay.

### Cell treatment

Cells were treated with DMSO, rapamycin (5 µM), CQ (50 µM), NH_4_Cl (10 mM), leupeptin (50 µM), CHX (100 µg/mL), MG132 (20 µM), or starvation for the indicated times before collection. The cells were then collected for subsequent experiments.

### RNA interference

Small interfering RNAs (siRNAs) against LAMP2A and negative control (siRNA-NC) were designed and synthesized by GenePharma (Shanghai, China). MARC-145 cells or PAMs were transfected with the indicated siRNAs at a final concentration of 50 nM using Lipofectamine 2000 Transfection Reagent for 24–48 h according to the manufacturer’s instructions. The transfected cells were used for subsequent experiments. The indicated siRNAs are listed in Table S2.

### Western blotting

Cells were harvested and lysed immediately in lysis buffer [2% sodium dodecyl sulfate (SDS), 1% Triton X-100 (Solarbio, T8200), 50 mM Tris-HCl, 150 mM NaCl, pH 7.5]. Proteins were separated *via* SDS‒polyacrylamide gel electrophoresis and the separated protein bands were blotted onto nitrocellulose membranes (Cytiva, 10600001). After blocking the membrane with 5% nonfat dry milk containing 0.1% Tween 20 (Solarbio, T8220) for 30 min at 37°C, the membranes were incubated with primary antibodies for 6 h at 4°C, followed by incubation with HRP-conjugated anti-mouse/rabbit IgG for 1 h at 37°C, after which the membrane was visualized using Amersham Image Quant 800 (Cytiva, Washington, USA).

### Indirect immunofluorescence assay

HEK293T, MARC-145, or 3D4/21 cells were plated on 35 mm glass-bottomed cell culture dishes at a suitable seeding density. Within 24 h, the cells were either transfected with the indicated recombinant vectors or infected with PRRSV Hn07-1 at an MOI of 1. The cells were treated with the indicated reagents or cultured in a starvation medium. After 24 h, the cells were fixed with 4% paraformaldehyde (Servicebio, G1101) for 10 min at room temperature and then blocked with 5% nonfat milk for 1 h. After the cells were washed three times with phosphate-buffered saline containing Tween 20 (PBST), they were incubated with the indicated antibodies. The cells were then washed with PBST and incubated with FITC, Dylight 405, Alexa Fluor 546, or Alexa Fluor 647-labeled IgG antibodies for 1 h. After the cells were washed three times with PBST, they were incubated with DAPI. The cells were washed with phosphate-buffered saline (PBS) and then observed under a Zeiss LSM 800 laser scanning confocal microscope (Zeiss, Oberkochen, Germany).

### Co-immunoprecipitation (Co-IP) assay

HEK293T cells were separately cotransfected with the indicated recombinant vectors and then lysed using NP-40 lysis buffer containing PMSF (1 mM). The cell lysates were incubated with anti-Flag M2 affinity gel for 4 h at 4°C. After centrifugation, the supernatant was removed, and the pellets were suspended in a washing buffer. The centrifugation and resuspension processes were performed five times. Finally, the pellets were lysed in lysis buffer for western blotting analysis.

### Dual luciferase assay

HEK293T cells were cotransfected with pIFN-β-luc, pRL-TK, and Flag-NSP11 for 24 h, and then the cells were infected with SeV (50 HAU) for 12 h. The cells were lysed, and firefly luciferase and Renilla luciferase activities were measured using a dual luciferase reporter gene assay kit (Beyotime, RG028) according to the manufacturer’s protocol. The relative levels of firefly luciferase activity were standardized to those of pRL-TK.

### Statistical analysis

For quantitative analysis of GFP-LC3 puncta formation, only cells with at least five GFP dots or ring-like structures were scored as positive. The protein bands were quantified by western blotting analysis. Briefly, the mean gray value of protein bands within the linear range and the background were measured using ImageJ 1.46r software, and the quantification values will reflect the relative amounts as a ratio of each net band value relative to the net loading control. All the statistical analyses were performed with GraphPad Prism 8 software. Data are presented as the mean ± SD from three independent samples. Statistically significant differences between groups were determined using the Student’s *t*-test. *P* < 0.05 indicated statistical significance.

## RESULTS

### PRRSV infection could induce incomplete autophagy

**Fig 1:**
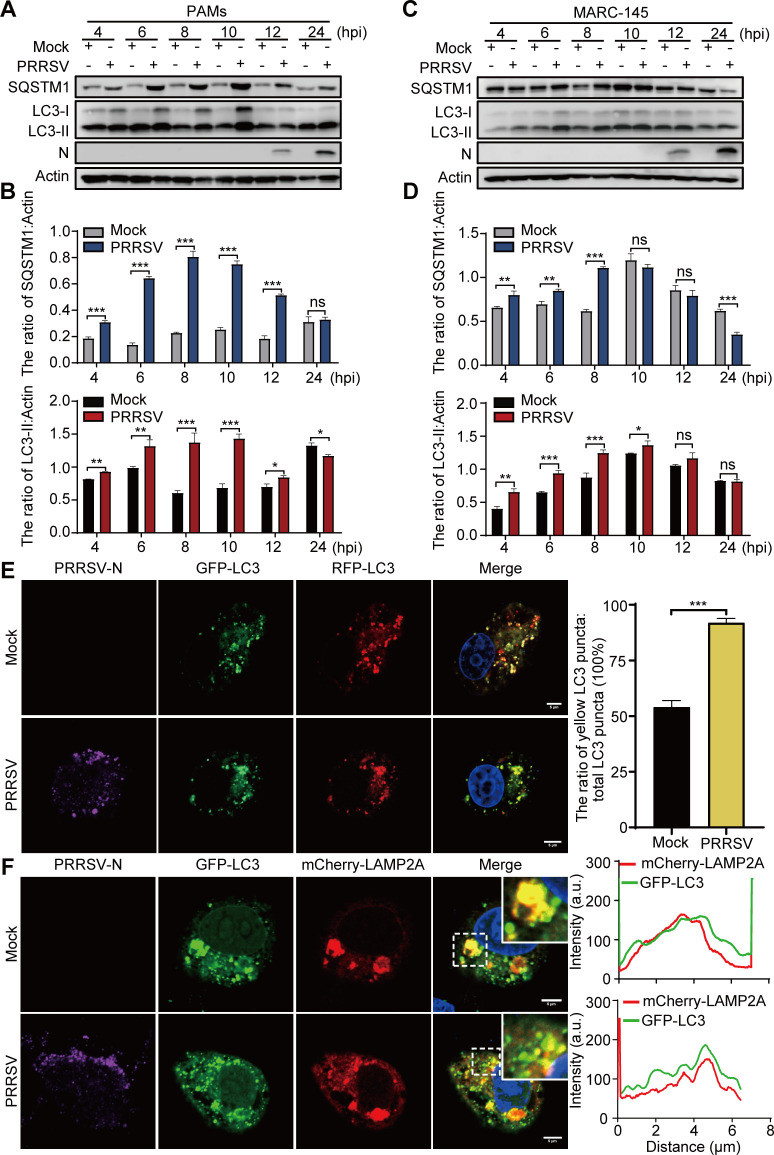
PRRSV infection induces incomplete autophagy. (**A, C**) PAMs (**A**) or MARC-145 cells (**C**) were infected with PRRSV at an MOI of 1 for the indicated times. The resultant cell lysates were subjected to immunoblotting using the indicated antibodies. (**B, D**) The ratio of LC3-II or SQSTM1 to Actin was normalized to control conditions in (**A**) or (**C**). (**E, F**) MARC-145 cells, which were pre-transfected with RFP-GFP-LC3 (**E**), or GFP-LC3 and mCherry-LAMP2A (**F**) for 24 h, were infected with PRRSV at an MOI of 1 for 8 h. Cells were treated with rapamycin (5 µM) for 6 h before collection. The cells were immunostained with anti-PRRSV-N antibody and observed under a confocal microscope. The scale bar indicates 5 µm. Error bars: mean ± SD of 3 independent tests. Student’s *t*-test; ns: non-significance; *P < 0.05; **P < 0.01; ***P < 0.001 compared to control.

In addition to PAMs, PRRSV is strictly cytotropic to African green monkey kidney cells, such as MARC-145 cells. To explore the underlying mechanisms by which PRRSV modulates autophagic flux, we first examined the changes in the expression of LC3, an autophagosome marker, in PAMs and MARC-145 cells infected with PRRSV [multiplicity of infection (MOI )=1] at 4–24 h post-infection (hpi). Moreover, the accumulation of sequestosome 1 (SQSTM1), a substrate of autophagy, was detected to evaluate the degradation function of autophagy. As shown in Fig. 1A and B, the levels of intracellular LC3-II and SQSTM1 were significantly greater at 4–12 hpi in PRRSV-infected PAMs than in mock infection. Similarly, LC3-II conversion and SQSTM1 accumulation were also upregulated in MARC-145 cells at 4–8 hpi (Fig. 1C and D). These results indicate that PRRSV infection is beneficial for the formation of autophagosomes but disadvantageous for the degradation of autophagy.

The increase in the intracellular LC3-II content can be caused either by an increase in autophagosome formation or the blockade of autophagic flux, whereas SQSTM1 accumulation usually results from reduced degradation due to autophagic flux blockade. To investigate which of these two mechanisms contributed to autophagosome accumulation upon PRRSV infection, we analyzed whether PRRSV infection affected the fusion of autophagosomes and lysosomes. The tandem reporter construct RFP-GFP-LC3 was transfected into uninfected or PRRSV-infected MARC-145 cells. The green fluorescent protein (GFP) moiety of this tandem autophagosome marker is sensitive to lysosomal proteolysis and quenching at acidic pH, whereas red fluorescent protein (RFP) is not sensitive. Therefore, the green fluorescent component of the RFP-GFP-LC3 reporter is lost during autophagosome fusion with lysosomes (27). As shown in Fig. 1E, compared with mock infection, PRRSV infection promoted the accumulation of RFP and GFP double-positive vesicles (yellow puncta), suggesting impaired fusion of autophagosomes and lysosomes. Furthermore, the colocalization of the autophagosome marker LC3 with the lysosome marker LAMP2A was analyzed. As shown in Fig. 1F, compared with mock infection, PRRSV infection markedly increased the numbers of total LC3 puncta but decreased the colocalization signals of LC3 and LAMP2A. It is indicated that PRRSV infection could induce the formation of autophagosomes but inhibit the fusion of autophagosomes and lysosomes. In addition, PRRSV infection induced normal autophagic flux (Fig. S1A and B). Moreover, PRRSV infection with either rapamycin (an autophagy inducer) or CQ (an autophagy inhibitor) promoted the accumulation of RFP and GFP double-positive vesicles and decreased the colocalization signals of LC3 and LAMP2A (Fig. S1C and D). Taken together, these results suggest that PRRSV infection could induce incomplete autophagy.

### GP5 is the main regulator of PRRSV-induced autophagy

**Fig 2:**
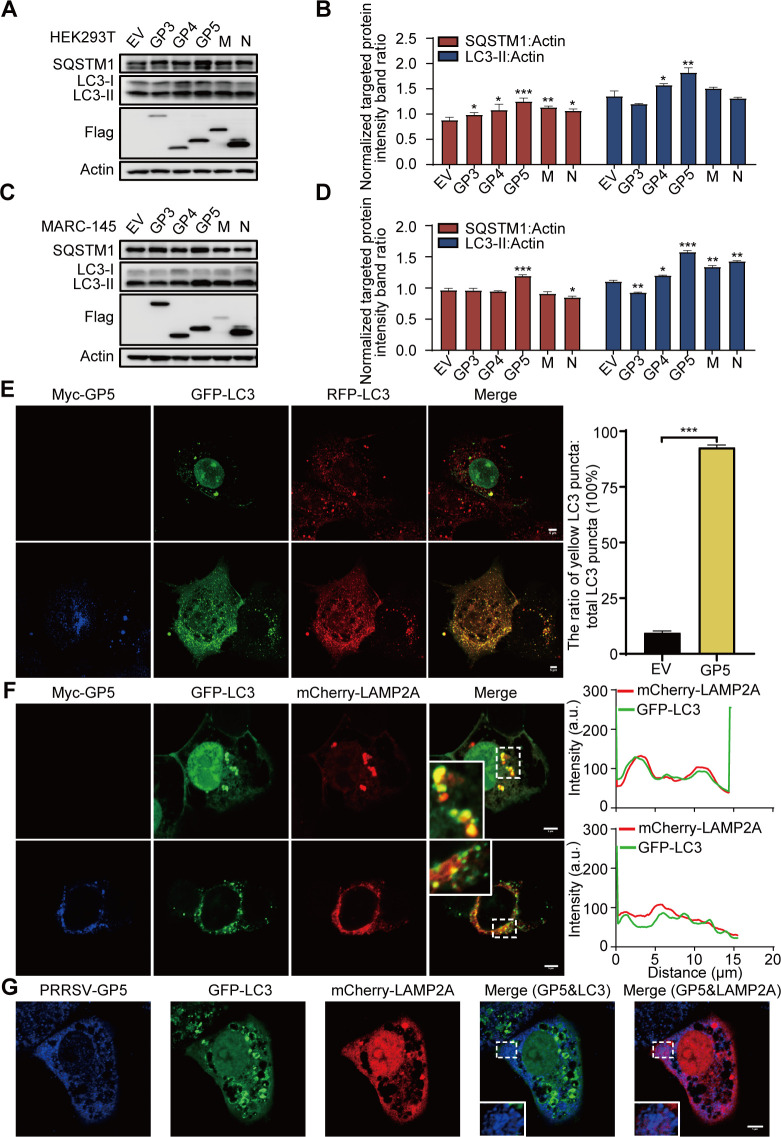
GP5 is the main regulator of PRRSV-induced autophagy. (**A, C**) HEK293T (**A**) or MARC-145 cells (**C**) were transfected with vectors expressing GP3, GP4, GP5, M, or N. The resultant cell lysates were subjected to immunoblotting using the indicated antibodies. (**B, D**) The ratio of SQSTM1 or LC3-II to Actin was normalized to control conditions in (**A**) or (**C**). (**E, F**) 3D4/21 cells were cotransfected with Myc-GP5 and RFP-GFP-LC3 (**E**), or Myc-GP5, GFP-LC3, and mCherry-LAMP2A (**F**). Cells were treated with rapamycin (5 µM) for 6 h before collection. The cells were then immunostained with anti-Myc pAb and observed under a confocal microscope. (**G**) MARC-145 cells, which were pre-transfected with GFP-LC3 and mCherry-LAMP2A for 24 h, were infected with PRRSV at an MOI of 1 for 8 h. Cells were treated with CQ (50 µM) for 6 h before collection. The cells were immunostained with anti-PRRSV-GP5 antibody and observed under a confocal microscope. EV: Empty vector. The scale bar indicates 5 µm. Error bars: mean ± SD of 3 independent tests. Student’s *t*-test; *P < 0.05; **P < 0.01; ***P < 0.001 compared to control.

To investigate how PRRSV causes this drastic change in the autophagic activity of infected cells, we explored the viral proteins responsible for blocking autophagy. HEK293T or MARC-145 cells were transfected with vectors expressing GP3, GP4, GP5, M, or N, and we examined the conversion of endogenous LC3 I to LC3 II and the accumulation of SQSTM1. The results showed that overexpression of GP5 significantly promoted the formation of LC3-II and increased the protein levels of SQSTM1 (Fig. 2A through D). As shown in Fig. 2E, GP5 might lead to the accumulation of RFP and GFP double-positive vesicles (yellow puncta), suggesting a blocked fusion of autophagosomes and lysosomes. Furthermore, GP5 obviously decreased the colocalization of LC3 and LAMP2A (Fig. 2F). These results show that GP5 is the main inhibitor of PRRSV-induced autophagy. However, we found that GP5 might have a greater binding affinity for LAMP2A than LC3 (Fig. 2F and G). Thus, we speculated that the GP5-mediated inhibition of autophagy might be mainly related to the lysosome.

### GP5 interacts with CMA key protein HSC70 and LAMP2A

**Fig 3:**
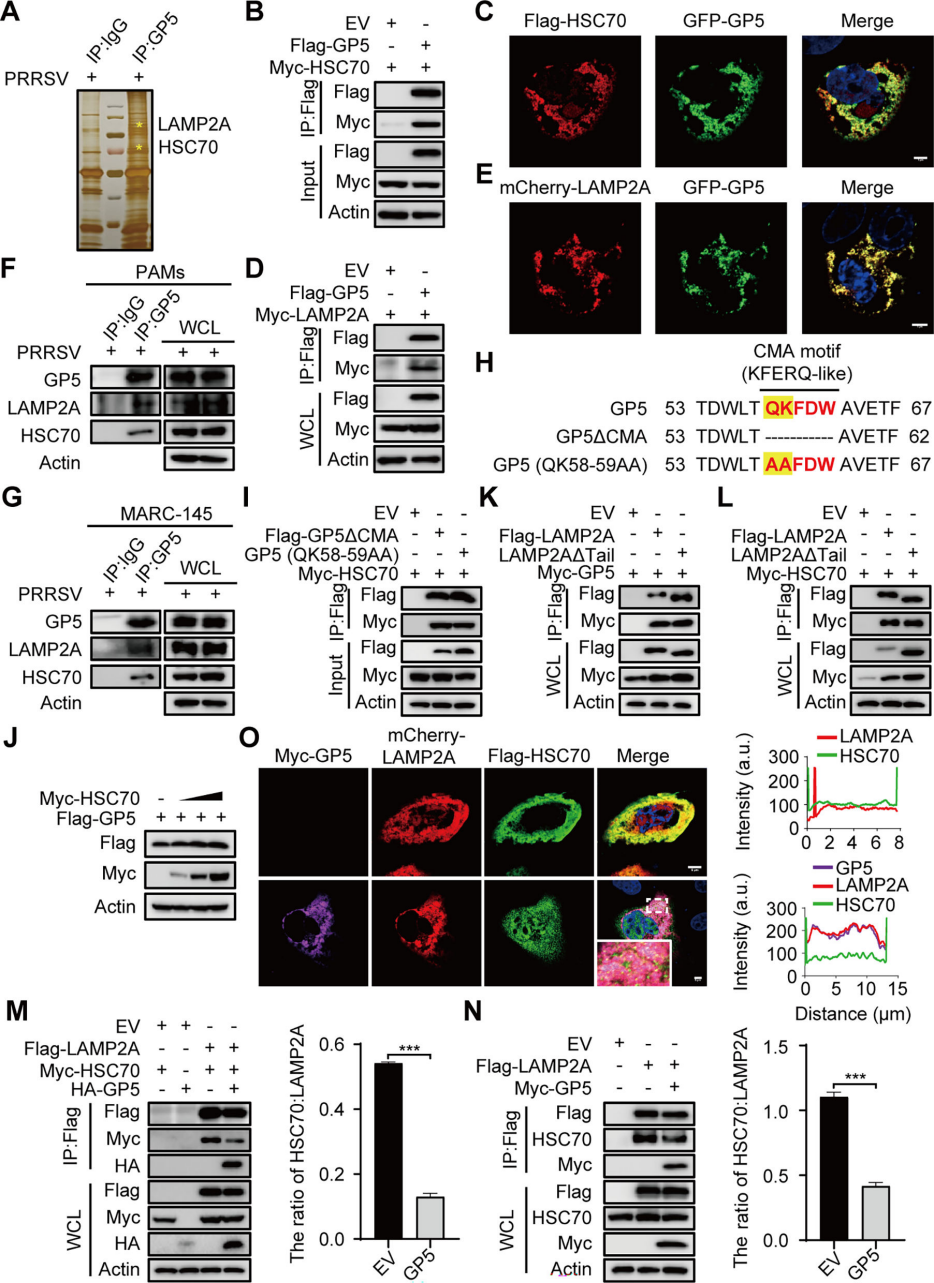
GP5 disrupts the interaction between LAMP2A and HSC70 by competitively binding to LAMP2A. (**A**) PAMs were infected with PRRSV at an MOI of 1 for 36 h, and a co-IP assay was performed by the anti-PRRSV-GP5 antibody. GP5-interacting host proteins were eluted and analyzed on SDS-PAGE followed by silver staining. *: HSC70 or LAMP2A. (**B, D**) HEK293T cells were cotransfected with Flag-GP5 and Myc-HSC70 (**B**), or Flag-GP5 and Myc-LAMP2A (**D**) for 24 h. The cell lysates were immunoprecipitated with anti-Flag IgG beads and subjected to immunoblotting using the indicated antibodies. (**C, E**) 3D4/21 cells were cotransfected with GFP-GP5 and Flag-HSC70 (**C**), or GFP-GP5 and mCherry-LAMP2A (**E**) for 24 h. Cells were immunostained with anti-Flag mAb and observed under a confocal microscope. (**F, G**) PAMs (**F**) and MARC-145 cells (**G**) were infected with PRRSV at an MOI of 1 for 36 h, and a co-IP assay was performed by the anti-PRRSV-GP5 antibody. GP5-interacting host proteins were eluted and analyzed on immunoblotting. (**H**) Schematic representation of GP5 mutants. The CMA motif was marked in red. The mutant regions were marked in yellow. (**I**) HEK293T cells were cotransfected with vectors expressing GP5 mutants and Myc-HSC70 for 24 h. The cell lysates were immunoprecipitated with anti-Flag IgG beads and subjected to immunoblotting using the indicated antibodies. (**J**) HEK293T cells were transfected with Flag-GP5 (2 µg) and increasing amounts of Myc-HSC70 (0–2 μg) for 24 h. The cell lysates were then subjected to immunoblotting using the indicated antibodies. (**K, L**) HEK293T cells were cotransfected with Flag-LAMP2AΔTail and Myc-GP5 (**K**) or Flag-LAMP2AΔTail and Myc-HSC70 (**L**) for 24 h. The cell lysates were subjected to analysis of Flag-precipitation and immunoblotting analysis. (**M, N**) HEK293T cells were cotransfected with Flag-LAMP2A, Myc-HSC70, and HA-GP5 for 24 h (**M**). HEK293T cells were cotransfected with Flag-LAMP2A and Myc-GP5 for 24 h (**N**). The cell lysates were subjected to analysis of Flag-precipitation and immunoblotting analysis. (**O**) 3D4/21 cells were transfected with Myc-GP5, Flag-HSC70, and mCherry-LAMP2A for 24 h. Cells were immunostained with anti-Myc pAb and anti-Flag mAb and observed under a confocal microscope. WCL: whole cell lysate. The scale bar indicates 5 µm. Error bars: mean ± SD of 3 independent tests. student’s *t*-test; ***P < 0.001 compared to control.

To further explore the inhibitory effect of GP5 on autophagy, we performed a co-IP assay combined with liquid chromatography-mass spectrometry (LC-MS/MS) in PAMs. The cell lysates were examined by SDS-polyacrylamide gel electrophoresis followed by silver staining. As shown in Fig. 3A, distinct bands could be observed in the lane of IP-GP5 on polyacrylamide gel compared to the negative control. Moreover, the LC-MS/MS results showed that HSC70 and LAMP2A were GP5-interacting proteins (Fig. S2A and B). Then, we evaluated the protein-protein interactions using co-IP assay and indirect immunofluorescence assay (IFA). As shown in Fig. 3B and D, GP5 could interact with HSC70 or LAMP2A. Furthermore, GP5 colocalized with HSC70 and LAMP2A (Fig. 3C and E) Consistently, we also verified an endogenous interaction between GP5 and HSC70 or LAMP2A (Fig. 3F and G). The above results confirmed that GP5 interacts with both HSC70 and LAMP2A.

When a protein interacts with both HSC70 and LAMP2A, it may be a substrate for CMA. The pentapeptide KFERQ-like motif is a necessary sequence in substrates, which is required for HSC70 to recognize and deliver to the lysosome receptor LAMP2A to initiate the degradation of CMA (28). Therefore, to confirm whether GP5 is a substrate of CMA, we analyzed the amino acid sequence of GP5 and found that GP5 exhibited the canonical KFERQ-like motif “QKFDW” (Fig. 3H). Then, we constructed two GP5 mutant vectors, GP5 (QK58-59AA) (58QK59 mutated to 58AA59) and GP5ΔCMA (58QKFDW62 deleted) (Fig. 3H), and each mutant was cotransfected with Myc-HSC70 into HEK293T cells. Interestingly, these GP5 mutants could still interact with HSC70 (Fig. 3I), and HSC70 promoted GP5 expression instead of degradation (Fig. 3J). According to the current studies, the substrate-HSC70 complex is recruited to the cytoplasmic tail of LAMP2A (12, 29). To figure out the phenomenon, the cytoplasmic tail-deleted LAMP2A mutant (LAMP2AΔTail) was constructed and cotransfected with Myc-GP5 or Myc-HSC70. As shown in Fig. 3K and L, the LAMP2A mutation could still interact with GP5 and HSC70. These results indicate that GP5 is not a substrate for CMA and that other domains that interact between HSC70 and GP5 may exist.

Next, we predicted the GP5 domains using online software (https://services.healthtech.dtu.dk/services/TMHMM-2.0/). The prediction results showed that GP5 contains one signal peptide, two extracellular regions, two intracellular regions, and three transmembrane regions. Subsequently, GP5 mutant vectors were constructed and cotransfected together with Myc-HSC70. We then examined the interaction between GP5 mutants and HSC70 by performing IFA and co-IP assay. The N-terminal 66–88 and 126–201 amino acid regions of GP5 were found to mediate the interaction between GP5 and HSC70 (Fig. S2C through E). It is confirmed that the “QKFDW” motif of GP5 is not the region where GP5 interacts with HSC70.

GP5 interacts with both HSC70 and LAMP2A, so we further studied their interactions. Co-IP assay revealed that GP5 disrupted the interaction between LAMP2A and HSC70 by competitively binding to LAMP2A (Fig. 3M and N). Furthermore, confocal microscope analysis revealed that the colocalization of HSC70 and LAMP2A was evidently weaker in the presence of GP5 (Fig. 3O). It has been reported that the two-domain architecture of the Lumenal domains in LAMP2A underlies the interaction with HSC70, especially the membrane-distal region within the Lumenal domain (30). Subsequently, we constructed domain-deleted mutants of LAMP2A (Fig. S3A) to confirm its interaction regions. As shown in Fig. S3B and C, deletion of the Hinge or Lumenal 1 domain disrupted the interaction between LAMP2A and HSC70, while deletion of the Hinge or TM domain disrupted the interaction between LAMP2A and GP5 in the co-IP assay. Taken together, these results indicate that GP5 competitively binds to the Hinge domain of LAMP2A and disrupts the formation of the LAMP2A-HSC70 complex.

### GP5 decreases the activity of CMA by inhibiting the MTORC2/PHLPP1/GFAP pathway

**Fig 4:**
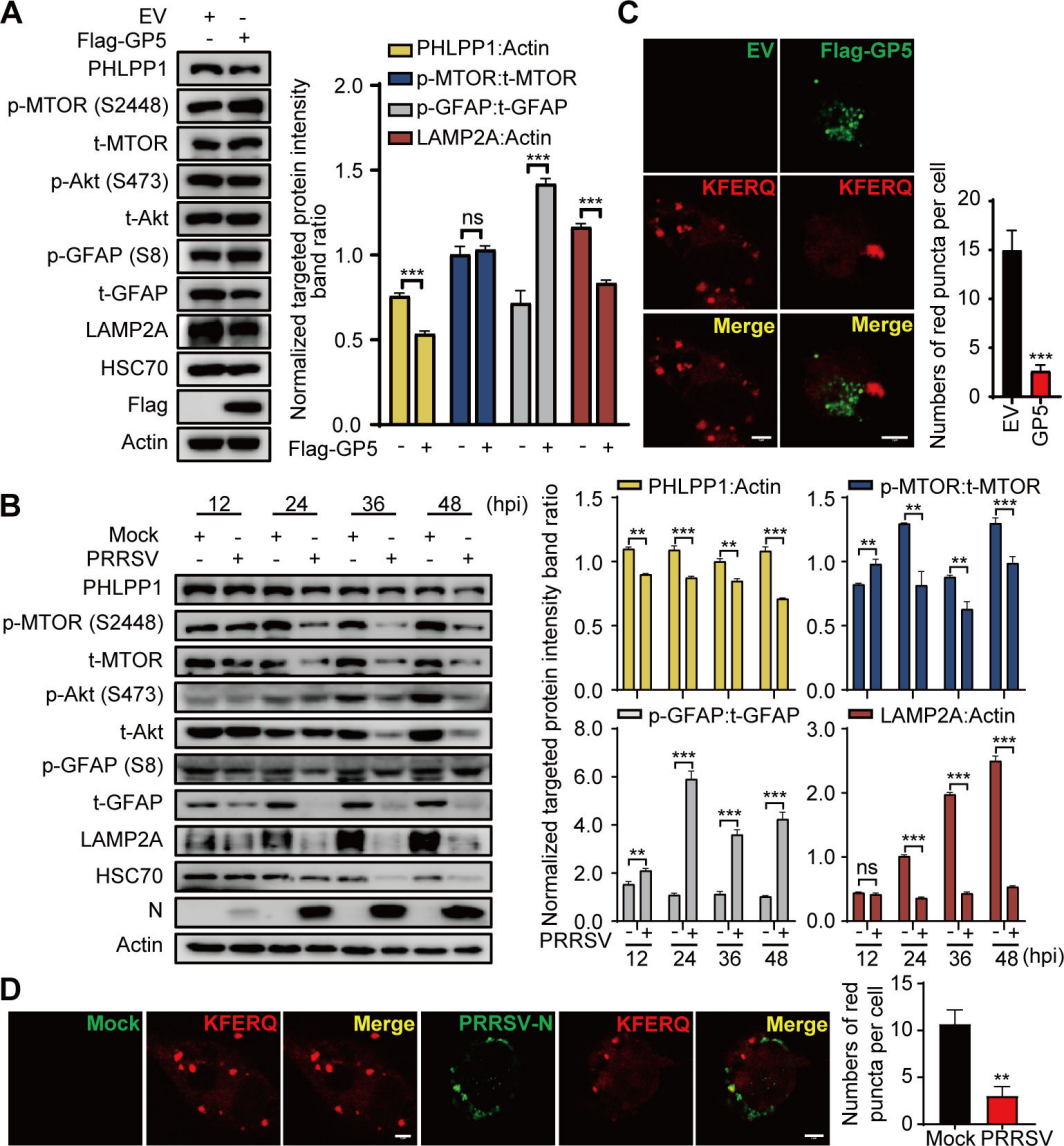
GP5 decreases the activity of CMA. (**A**) MARC-145 cells were transfected with Flag-GP5 for 36 h. Cells were then subjected to immunoblotting using the indicated antibodies. (**B**) PAMs were infected with PRRSV at an MOI of 1 for the indicated times. Cells were then subjected to immunoblotting using the indicated antibodies. (**C**) MARC-145 cells were cotransfected with Flag-GP5 and KFERQ-PA-mCherry for 36 h. Cells were immunostained with anti-Flag mAb, and then observed under a confocal microscope. (**D**) MARC-145 cells, which were pre-transfected with KFERQ-PA-mCherry, were infected with PRRSV for 24 h. Cells were immunostained with anti-PRRSV-N antibody and then observed under a confocal microscope. The scale bar indicates 5 µm. Error bars: mean ± SD of 3 independent tests. Student’s *t*-test; ns: non-significance; **P < 0.01; ***P < 0.001 compared to control.

Since GP5 is not a substrate for CMA but competitively binds to LAMP2A, we speculated that GP5 might regulate the activity of CMA by interacting with LAMP2A. Subsequently, we further explored the levels of key CMA proteins in cells with GP5 overexpression or PRRSV infection. As shown in Fig. 4A; Fig. S4A, GP5 downregulated the protein levels of PHLPP1, GFAP, and LAMP2A and upregulated the phosphorylation of GFAP in MARC-145 cells. Similarly, PRRSV infection decreased the protein levels of PHLPP1, GFAP, and LAMP2A and promoted the phosphorylation of GFAP in PAMs and MARC-145 cells (Fig. 4B; Fig. S4B). These results indicate that GP5 could decrease the activity of CMA by inhibiting the MTORC2/PHLPP1/GFAP pathway.

Moreover, increased levels of known CMA substrates, such as GAPDH, ribonuclease A, and alpha-synuclein, in isolated lysosomes are a good indication of CMA activation (31). Therefore, we evaluated CMA activity in GP5-transfected MARC-145 cells with the photoswitchable artificial substrate KFERQ-PA-mCherry, a photoactive CMA reporter that tracks lysosomal uptake. KFERQ-PA-mCherry is initially nonfluorescent but exhibits red fluorescence upon irradiation with 405 nm visible light, allowing visualization of lysosomes as red fluorescent puncta. The number of red puncta per cell is a reliable indicator of CMA (32). As shown in Fig. 4C and D, the number of red fluorescent puncta decreased in MARC-145 cells transfected with GP5 or infected with PRRSV. Collectively, these data demonstrated that PRRSV could decrease the activity of CMA through GP5.

### GP5 accelerates the disassembly of LAMP2A by dissociating the unmodified GFAP-LAMP2A complex

**Fig 5:**
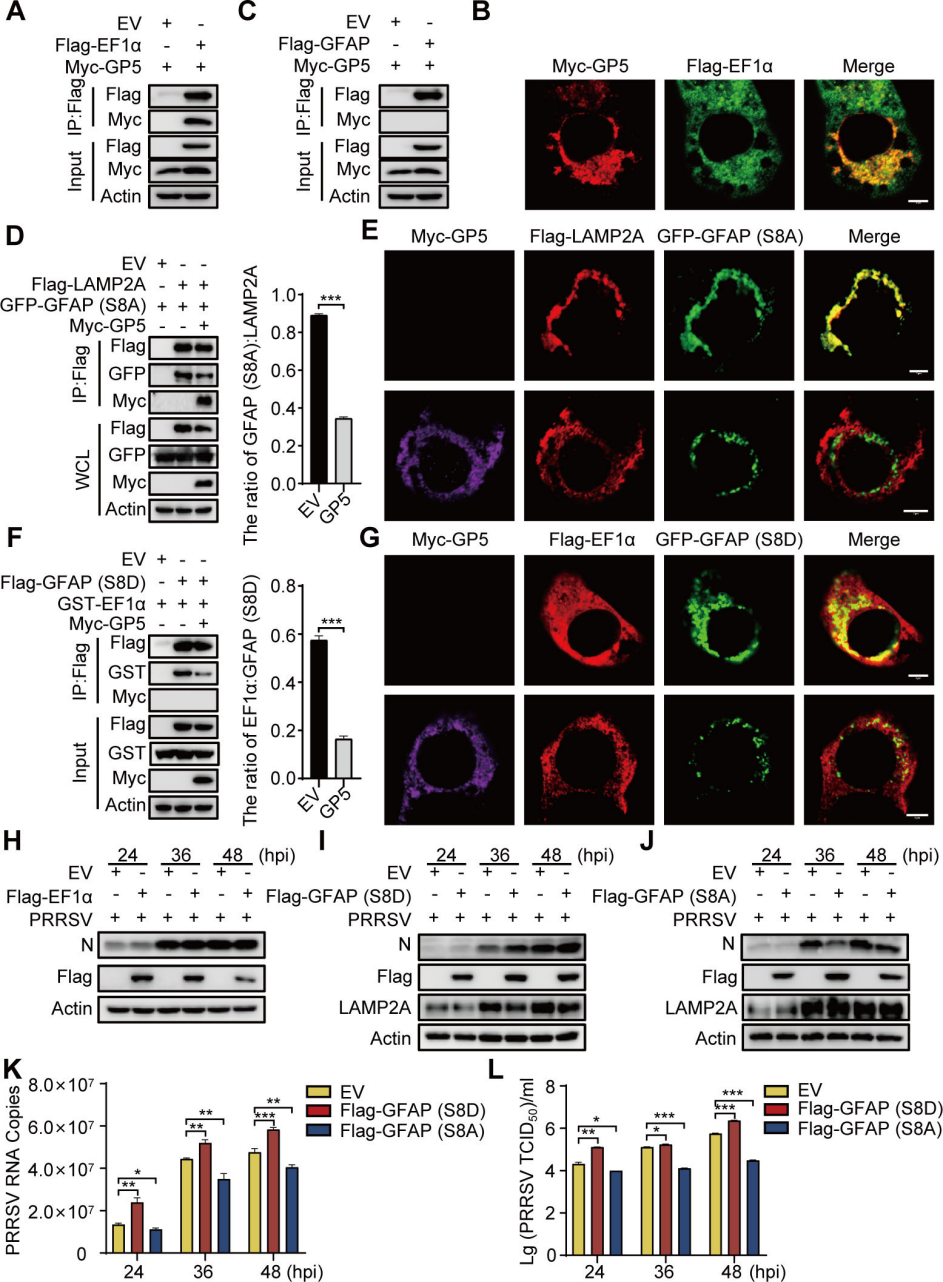
GP5 dissociates the unmodified GFAP-LAMP2A complex. (**A, C**) HEK293T cells were cotransfected with Myc-GP5 and Flag-EF1α (**A**), or Myc-GP5 and Flag-GFAP (**C**) for 24 h. The cell lysates were subjected to analysis of Flag-precipitation and immunoblotting analysis. (**B**) MARC-145 cells were cotransfected with Myc-GP5 and Flag-EF1α for 24 h. Cells were immunostained with anti-Myc pAb and anti-Flag mAb and observed under a confocal microscope. (**D, F**) HEK293T cells were cotransfected with Flag-LAMP2A, GFP-GFAP (S8A) and Myc-GP5 (**D**), or Flag-GFAP (S8D), GST-EF1α, and Myc-GP5 (**F**). The cell lysates were subjected to analysis of Flag-precipitation and immunoblotting analysis. (**E, G**) MARC-145 cells were cotransfected with Myc-GP5, Flag-LAMP2A, and GFP-GFAP (S8A) (**E**), or Myc-GP5, Flag-EF1α, and GFP-GFAP (S8D) (**G**) for 24 h. Cells were immunostained with anti-Myc pAb and anti-Flag mAb and then were observed under a confocal microscope. (**H**) MARC-145 cells, which were pre-transfected with Flag-EF1α, were infected with PRRSV at an MOI of 1 for the indicated times. The cellular extracts were then subjected to western blotting using the indicated antibodies. (**I-L**) MARC-145 cells, which were pre-transfected with Flag-GFAP (S8D) or Flag-GFAP (S8A), were infected with PRRSV at an MOI of 1 for the indicated times. The cells were harvested for western blotting (**I, J**) and RT-qPCR (**K**), and supernatants were collected for TCID_50_ measurement (**L**). The scale bar indicates 5 µm. Error bars: mean ± SD of 3 independent tests. Student’s *t*-test; *P < 0.05; **P < 0.01; ***P < 0.001 compared to control.

As shown in Fig. 4A, GP5 could upregulated the phosphorylation of GFAP and downregulated the expression of LAMP2A, we thus guessed that this may be a key factor in the disruption of CMA by GP5. Then, we detected the effect of GP5 on other CMA regulators. Co-IP assay and IFA revealed that GP5 interacted with EF1α rather than GFAP (Fig. 5A through C). In addition, by transfecting HEK293T cell with Flag-LAMP2A and GFAP-dephosphorylated mutant vector GFP-GFAP (S8A), we observed a decreased interaction of LAMP2A with GFAP (S8A) in the presence of GP5 (Fig. 5D and E). Further research showed that GP5 disrupted the interaction between the GFAP phosphomimetic mutant GFAP (S8D) and EF1α (Fig. 5F and G). These results confirmed that GP5 increases the dissociation of the pGFAP-EF1α complex and drives unmodified GFAP away from multimerized LAMP2A, resulting in the disassembly of LAMP2A and decreasing the activity of CMA.

In addition, to evaluate the effects of GFAP on the replication of PRRSV, MARC-145 cells, which were pre-transfected with Flag-EF1α, Flag-GFAP (S8D), or Flag-GFAP (S8A), were infected with PRRSV for 24–48 h. Then, the cells were harvested for western blotting and RT-qPCR analysis, and the supernatants were collected for 50% tissue culture infective dose (TCID_50_) measurement. We found that EF1α had no significant effect on PRRSV multiplication (Fig. 5H), but GFAP (S8D) reduced the expression of LAMP2A, resulting in increased N protein levels and PRRSV genome copies in the cell lysates (Fig. 5I and K) and increased viral yield in the culture supernatant (Fig. 5L). Conversely, GFAP (S8A) could maintain the stability of LAMP2A and reduce PRRSV N protein levels, PRRSV genome copies, and viral yield (Fig. 5J through L). Therefore, we speculated that the activity of CMA might affect PRRSV proliferation.

### GP5 impairs the stability of LAMP2A by blocking its K63-linked polyubiquitination

**Fig 6:**
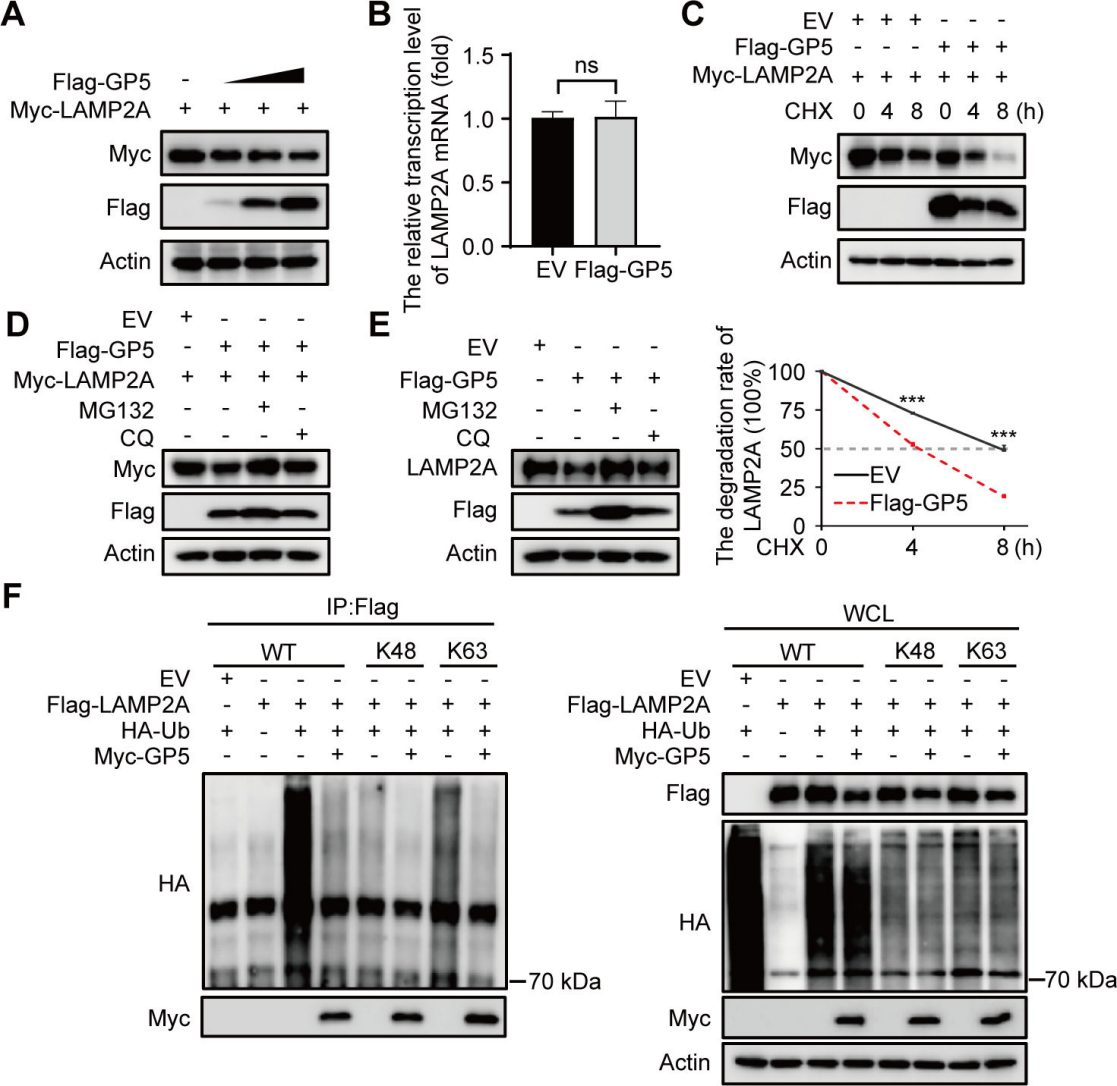
GP5 impairs the protein stability of LAMP2A by UPS. (**A**) HEK293T cells were cotransfected with Myc-LAMP2A (2 µg) and increasing amounts of Flag-GP5 (0–2 μg) for 24 h. The cellular extracts were then subjected to western blotting using the indicated antibodies. (**B**) HEK293T cells were transfected with EV pCMV-Flag or Flag-GP5 for 24 h. The cellular extracts were then subjected to RT-qPCR. (**C**) HEK293T cells were cotransfected with Myc-LAMP2A and Flag-GP5, then treated with CHX (100 µg/mL) for the indicated times after 24-h transfection. The cell samples were subjected to immunoblotting analysis with the indicated antibodies. (**D, E**) HEK293T cells were cotransfected with Myc-LAMP2A and Flag-GP5 for 24 h (**D**). MARC-145 cells were transfected with Flag-GP5 for 36 h (**E**). Cells were treated with MG132 (20 µM) or CQ (50 µM) for 6 h before collection. The cellular extracts were then subjected to western blotting using the indicated antibodies. (**F**) HEK293T cells were cotransfected with Flag-LAMP2A, HA-Ub (Ub: ubiquitin, WT: wild-type, K at indicated residue, and K at other residues were simultaneously mutated to arginines), and Myc-GP5 for 24 h. Cells were treated with MG132 (20 µM) for 6 h before collection. The cell lysates were subjected to analysis of Flag-precipitation and immunoblotting analysis. The scale bar indicates 5 µm. Error bars: mean ± SD of 3 independent tests. Student’s *t*-test; ns: non-significance; ***P < 0.001 compared to control.

Since LAMP2A has an important effect on CMA activity, the above results showed that GP5 could reduce the protein levels of LAMP2A (Fig. 4A and 5D ). Therefore, we next further explored how GP5 induces LAMP2A degradation. First, we cotransfected HEK293T cells with Myc-LAMP2A and a gradient of increasing amounts of Flag-GP5. The western blotting results showed that the protein levels of LAMP2A decreased in a GP5 dose-dependent manner (Fig. 6A). However, the RT-qPCR results showed that there was no significant change in the LAMP2A mRNA transcript level (Fig. 6B), suggesting that GP5 may abrogate the stability of LAMP2A. Subsequently, western blotting analysis indicated that GP5 overexpression decreased the LAMP2A half-life (Fig. 6C), demonstrating that GP5 may accelerate LAMP2A degradation. These results show that GP5 could promote LAMP2A degradation by reducing its stability.

Protein degradation predominantly occurs through two pathways: a proteasome-dependent pathway and a lysosome-dependent pathway. To investigate the mechanism of GP5-mediated LAMP2A degradation, we cotransfected HEK293T cells with Flag-GP5 and Myc-LAMP2A and then treated the cells with either a proteasome inhibitor (MG132) or a lysosomal inhibitor (CQ). The western blotting results showed that Myc-LAMP2A could be mostly restored in the cells by treating the cells with MG132 but not with CQ (Fig. 6D). Consistently, in GP5-expressing MARC-145 cells, treatment with MG132 but not CQ reversed the GP5-mediated inhibition of LAMP2A expression (Fig. 6E). This phenomenon suggested that GP5 promotes LAMP2A degradation through the ubiquitin-proteasome pathway. As the ubiquitin-proteasome system (UPS) plays an important role in regulating protein degradation, we subsequently determined whether GP5 mediates the polyubiquitination of LAMP2A. Among all the polyubiquitin linkages, K48 and K63 are the most abundant types of ubiquitin linkage in mammalian cells (33). We thus tested whether GP5 could affect the K48- or K63-linked polyubiquitination of LAMP2A. The results showed that GP5 overexpression markedly inhibited K63-linked polyubiquitination of LAMP2A (Fig. 5F). Consequently, these results indicate that GP5 could weaken the stability of LAMP2A by impairing K63-linked polyubiquitination, thereby promoting its degradation.

### GP5 inhibits the antiviral effect of CMA

**Fig 7:**
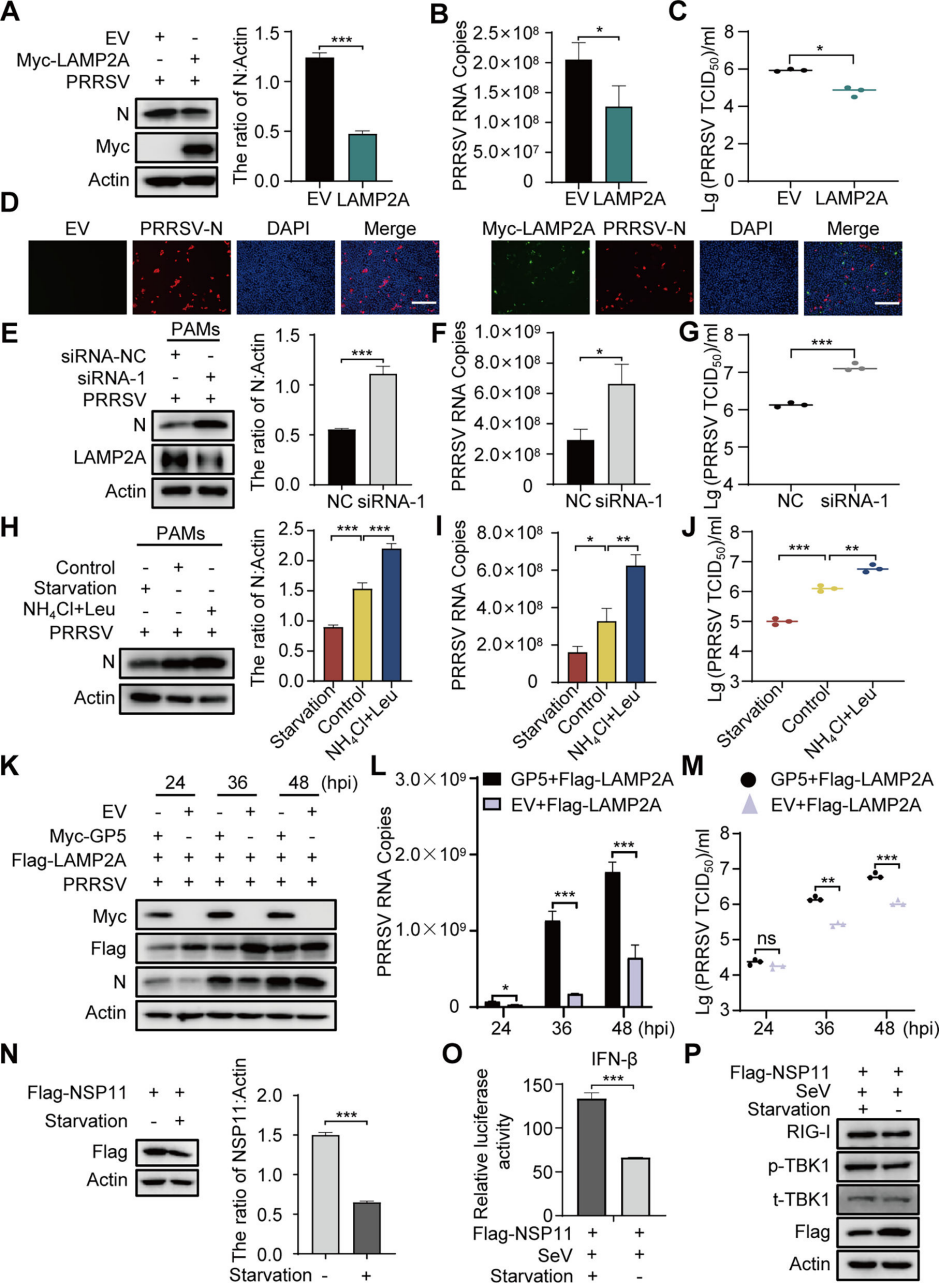
The activation of CMA inhibits PRRSV replication. (**A-D**) MARC-145 cells, which were pre-transfected with Myc-LAMP2A, were infected with PRRSV for 36 h. Cells were analyzed *via* western blotting (**A**), RT-qPCR (**B**), and IFA (**D**), and culture supernatants were collected for TCID_50_ measurement (**C**). (**E-G**) PAMs, which were pre-transfected with siRNA-LAMP2A, were infected with PRRSV for 36 h. Cells were harvested for western blotting (**E**) and RT-qPCR (**F**) analysis, and culture supernatants were collected for TCID_50_ measurement (**G**). (**H-J**) PAMs were infected with PRRSV for 24 h and then treated with starvation for 16 h or NH_4_Cl (10 mM) and leupeptin (Leu; 50 µM) for 12 h. Cells were harvested for western blotting (**H**) and RT-qPCR (**I**) analysis, and supernatants were collected for TCID_50_ measurement (**J**). (**K-M**) MARC-145 cells, which were pre-transfected with Myc-GP5 and Flag-LAMP2A, were infected with PRRSV at an MOI of 1 for the indicated times. The cells were harvested for western blotting (**K**) and RT-qPCR (**L**), and supernatants were collected for TCID_50_ measurement (**M**). (**N**) HEK293T cells were transfected with vectors expressing PRRSV viral proteins for 24 h and then treated with starvation for 16 h. The cell lysates were subjected to immunoblotting analysis. (**O**) HEK293T cells were cotransfected with pIFN-β-luc, pRL-TK, and Flag-NSP11. After 24 h, the cells were infected with SeV (50 HAU) for 2 h, and then treated with starvation for 16 h. The cells were analyzed by luciferase reporter assay system. (**P**) HEK293T cells were transfected with Flag-NSP11. After 24 h, the cells were infected with SeV (50 HAU) for 2 h, and then treated with starvation for 16 h. The cells were lysed and analyzed by western blotting. The scale bar indicates 300 µm. Error bars: mean ± SD of 3 independent tests. Student’s *t*-test; ns: non-significance; *P < 0.05; **P < 0.01; ***P < 0.001 compared to control.

Since GP5 could inhibit the CMA activity (Fig. 4) and the phosphorylation of GFAP affects PRRSV proliferation (Fig. 5), we further investigated the effect of CMA activity on PRRSV replication. First, we investigated the LAMP2A overexpression on PRRSV replication. The results showed that LAMP2A overexpression reduces PRRSV N protein levels and genome copies in the cell lysates and decreases the viral yield in the culture supernatant (Fig. 7A through D). Next, we designed two siRNAs to target LAMP2A. The knockdown efficiencies of the siRNAs were detected using western blotting. As shown in Fig. S5A, siRNA-1 had a significant knockdown effect in PAMs. However, siRNA-2 could knockdown LAMP2A in MARC-145 cells by more than 80% at 36–48 h post-transfection (hpt) (Fig. S5B and C). We found that LAMP2A knockdown in PAMs increased N protein levels (Fig. 7E) and PRRSV genome copies in the cell lysate (Fig. 7F) and increased viral yield in the culture supernatant (Fig. 7G). Similarly, LAMP2A knockdown in MARC-145 cells promoted PRRSV replication (Fig. S5D through G).

Previous studies have shown that starvation is one of the best characterized stimuli for CMA, and the highest CMA activity appears at 10–16 h (31, 34, 35). Thus, we treated PRRSV-infected PAMs with starvation (CMA activator) or NH_4_Cl and leupeptin (CMA inhibitor). Compared to the control, starvation reduces N protein levels and PRRSV genome copies in the cell lysates (Fig. 7H and I) and decreases the viral yield in the culture supernatant (Fig. 7J). Conversely, as shown in Fig. 7H through J, the PRRSV N protein levels, PRRSV genome copies, and viral yield were increased in NH_4_Cl- and leupeptin-treated cell lysates and culture supernatant. Similarly, activation or inhibition of CMA had the same regulatory effect on PRRSV proliferation in MARC-145 cells (Fig. S5H through J). These results indicate that the activation of CMA inhibits PRRSV replication, while the inhibition of CMA promotes PRRSV replication. Furthermore, GP5 could reduce the LAMP2A protein levels, increase N protein levels and PRRSV genome copies in cell lysates (Fig. 7K and L), and increase the viral yield in the culture supernatant (Fig. 7M). These results indicate that GP5 inhibits the activation of CMA by targeting LAMP2A to promote PRRSV replication.

To understand the anti-PRRSV mechanism of CMA, we used western blotting to screen for PRRSV viral proteins degraded by CMA. The results showed that the activation of CMA could significantly decrease the expression of NSP11 (Fig. 7N; Fig. S6A). Previous studies have shown that NSP11 can reduce the expression of IFN-β (36). We also verified that NSP11 could inhibit the activity of the IFN-β promoter and the expression of RIG-I (Fig. S6B and C). Then, we tested the effect of CMA on NSP11-mediated IFN-I signaling by dual luciferase assay and western blotting. The results revealed that the activity of the IFN-β promoter and the expression of RIG-I were upregulated significantly under starvation in NSP11-overexpressing cells (Fig. 7O and P), indicating that the activation of CMA could antagonize the NSP11-mediated inhibition of IFN-I signaling. Altogether, these findings show that GP5 could strengthen the inhibitory effect of NSP11 on RIG-I-mediated IFN-I signaling *via* CMA.

## DISCUSSION

Autophagy is an evolutionarily conserved catabolic cellular process that exerts antiviral activity during viral invasion. Nevertheless, some viruses have acquired the ability to inhibit autophagy to evade degradation and immune responses. Some other viruses induce autophagy but then hijack autophagosomes and use them as a replication site, or hijack the secretory autophagy pathway to promote the maturation and egress of virus particles, thereby increasing replication (37).

The strategies that viruses adopt to manipulate autophagy to benefit their own replication are classified as inhibition of autophagosome formation and inhibition of fusion between autophagosomes and lysosomes (37). In this study, we found that PRRSV induced incomplete autophagic flux by inhibiting autophagosome-lysosome fusion (Fig. 1). Subsequently, we identified that GP5 was mainly involved in the inhibition of the complete autophagy process (Fig. 2A through F). This suppression promoted the accumulation of autophagosomes (Fig. 2E and F). Therefore, it indicated that PRRSV induced incomplete autophagy mainly through GP5. Moreover, we found that GP5 might have a greater binding affinity for LAMP2A than LC3 (Fig. 2F and G). Thus, we speculated that the GP5-mediated inhibition of autophagy might be mainly related to lysosomes.

Then, we identified two CMA markers, HSC70 and LAMP2A, that interact with GP5 (Fig. 3A through G; Fig. S2A and B). Owing to this unique mechanism, CMA is different from macroautophagy, in which its substrates enter the lysosome. HSC70 recognizes substrate proteins with a pentapeptide KFERQ motif and delivers them to the lysosomal surface (13, 31). Briefly, the peptide motif depends on the physical properties of the amino acid residues, which have been identified as a combination of one or two positively charged residues, one negatively charged residue, a combination of one or two hydrophobic residues, and a glutamine (Q) (28). Interestingly, sequence analysis revealed that GP5 has a KFERQ-like motif, “58QKFDW62” (Fig. 3H), which is consistent with the CMA motif rule. To investigate whether GP5 is degraded through CMA, we mutated residue 58QK59 in GP5 to alanine (A). It has been reported that this kind of mutant can disrupt the interaction between substrate and HSC70 and block CMA-dependent protein degradation. For example, the Plin2 mutant (417VQ418 mutated to 417AA418) exhibits decreased colocalization with HSC70 that of WT-Plin2 (38). The EIF4A1 mutant (93QI94 mutated to 93AA94) is significantly less competent for degradation by CMA than is WT-EIF4A1 (39). However, our results suggested that GP5 (QK58-59AA) could still interact with HSC70 (Fig. 3I) and that GP5 (66-88) and GP5 (126-201) mediate the interaction between GP5 and HSC70 (Fig. S2C and E). Previous studies have shown that the substrate-chaperone complex docks at the cytosolic tail of LAMP2A (12, 29) and that the two-domain architecture of LAMP2A regulates its interaction with HSC70 (30). However, our results indicated that GP5 and HSC70 could still interact with the cytosolic tail-deleted LAMP2A mutation (Fig. 3K and L). Collectively, the above results demonstrated that GP5 is not a substrate for CMA.

Further results showed that GP5 disrupted the interaction between LAMP2A and HSC70 by competitively binding to the Hinge domain of LAMP2A (Fig. 3M through O; Fig. S3A through C), indicating that substrate translocation might be impeded by GP5. LAMP2A is a key functional protein involved in CMA activity (16, 29), and the MTORC2/PHLPP1/GFAP pathway is an important signaling pathway regulating CMA activity (19). Therefore, we detected the protein levels of LAMP2A and the key proteins in this pathway. The results showed that both GP5 overexpression and PRRSV infection downregulated the protein levels of PHLPP1, GFAP, and LAMP2A and upregulated the phosphorylation of GFAP (Fig. 4A and B; Fig. S4A and B). Therefore, we hypothesized that PRRSV might utilize GP5 to decrease the activity of CMA by competitively binding to LAMP2A.

Previous studies revealed that unmodified GFAP binds to LAMP2A and contributes stabilization (18). Our results showed that GP5 disrupted the interaction between LAMP2A and GFAP (S8A) (Fig. 5D and E). It is reported that unmodified GFAP has a greater affinity with pGFAP than that with LAMP2A, and the dissociation of pGFAP from the pGFAP-EF1α complex promotes the dissociation of unmodified GFAP from the unmodified GFAP-LAMP2A complex, which ultimately results in the disassembly of LAMP2A (18, 19). Our above results showed that GP5 could upregulate the expression of pGFAP (Fig. 4A); therefore, we next examined the effect of GP5 on the stability of the pGFAP-EF1α complexes. The results showed that GP5 could disrupt the interaction between GFAP (S8D) and EF1α (Fig. 5F and G). These results indicate that GP5 could inhibit CMA by decreasing the formation of the unmodified GFAP-LAMP2A complex.

To further understand GP5 and how to decrease the activity of CMA, we explored the degradation mechanism of LAMP2A. It is reported that the UPS and the autophagy-lysosome system are two major cellular degradation systems in eukaryotes (40). Our results confirmed that GP5 promotes LAMP2A degradation through a ubiquitin-proteasome pathway (Fig. 6D and E). Further results demonstrated that GP5 promotes the degradation of LAMP2A by inhibiting its K63-linked polyubiquitination but not K48-linked polyubiquitination (Fig. 6F). K48-linked polyubiquitination is strongly associated with protein degradation, and K63-linked polyubiquitination mainly focuses on protein modification (41). However, the E3 ubiquitin ligase TRAF6 promotes K63-linked polyubiquitination of Unc-51-like autophagy-activating kinase (ULK1), thereby enhancing its stability and function (42). TMEM189 decreases ULK1 protein levels by inhibiting the K63-linked polyubiquitin of ULK1 (43). This finding demonstrated that GP5 could decrease the activity of CMA by disrupting the K63-linked polyubiquitination of LAMP2A.

Previous studies have shown that viral infection is related to the CMA (44–47). Our data illustrated that the activation of CMA could inhibit PRRSV proliferation by CMA activator treatment and LAMP2A or GFAP (S8A) overexpression (Fig. 7H through J ;Fig. 7A through D and Fig. 5J through L). Instead, the inhibition of CMA promoted PRRSV proliferation by CMA inhibitor treatment, LAMP2A knockdown, and GFAP (S8D) overexpression (Fig. 7E through J and Fig. 5I, K and L). Furthermore, GP5 overexpression could reverse the inhibitory effect of CMA on PRRSV replication (Fig. 7K through M). These results suggested that GP5 could inhibit the antiviral effect of CMA by decreasing its activity. In addition, we found that activation of CMA could significantly decrease the expression of PRRSV NSP11 (Fig. 7N; Fig. S6A). A previous study suggested that NSP11 inhibits the activity of the IFN-β promoter and suppresses the expression of RIG-I and IFN-β (48, 49). Our results were consistent with them (Fig. S6B and C). Further research showed that the activation of CMA could antagonize the NSP11-mediated inhibition of IFN-I signaling (Fig. 7O and P). Therefore, we hypothesized that CMA exerts its anti-PRRSV effect by activating NSP11-mediated IFN-I signaling.

It has been reported that LAMP2A can promote autophagic flux (50) while preventing autophagosome-lysosome fusion by silencing LAMP2A increases the titer of Coxsackievirus (44). In this study, we found that GP5 inhibited the fusion of autophagosomes and lysosomes (Fig. 2) and promoted the degradation of LAMP2A (Fig. 4D and 6). Therefore, we surmised that PRRSV GP5 might induce incomplete autophagic flux by inhibiting CMA. However, further studies are required to clarify the underlying mechanism involved.

**Fig 8:**
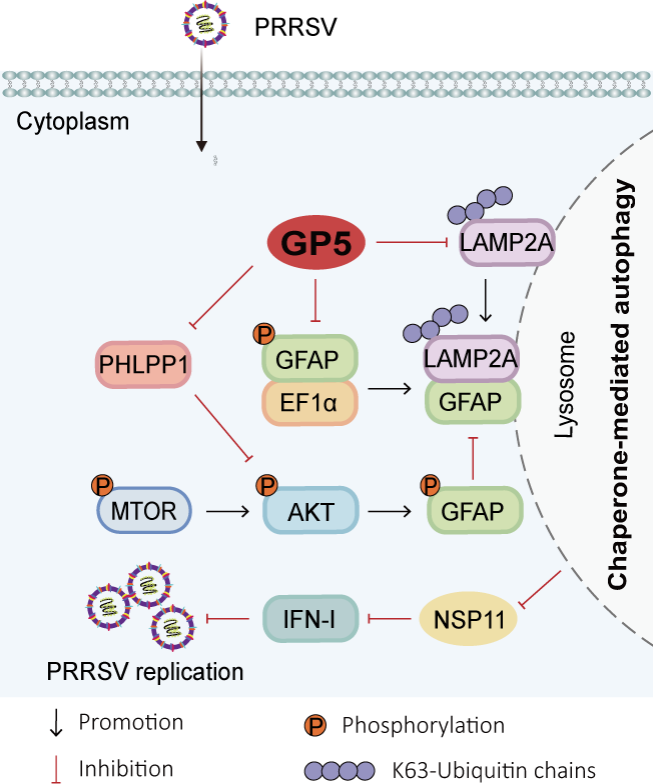
Schematic representation of the mechanism that GP5 inhibits the antiviral effects of CMA via targeting LAMP2A. The GFAP-LAMP2A complex promotes the activity of CMA and then antagonizes the inhibitory effect of the NSP11-mediated IFN-I signaling pathway to inhibit PRRSV replication. Conversely, PRRSV GP5 inhibits the MTORC2/PHLPP1/GFAP pathway, promotes the dissociation of the pGFAP-EF1α complex, and blocks the K63-linked polyubiquitination of LAMP2A, thus destroying the formation of the GFAP-LAMP2A complex and subsequently decreases the activity of CMA, and then strengthens the inhibitory effect of the NSP11-mediated IFN-I signaling pathway, therefore facilitates PRRSV replication.

In conclusion, we found that PRRSV GP5 inhibits the antiviral effect of CMA by targeting LAMP2A. Mechanistically, GP5 could decrease the activity of CMA by inhibiting the MTORC2/PHLPP1/GFAP pathway, destroying the interaction of the pGFAP-EF1α complex, and blocking the K63-linked polyubiquitination of LAMP2A, and then strengthen the inhibitory effect of the NSP11-mediated IFN-I signaling pathway, eventually promoting PRRSV replication (Fig. 8). This research provides new insight into the immune escape mechanism of immunosuppressive viruses in CMA.

## Data Availability

All relevant data are within the paper and its supplemental files.

## SUPPLEMENTAL MATERIAL

Supplemental figures mbio.00532-24-s0001.pdf Figures S1-S6.

Legends mbio.00532-24-s0002.docx Supplemental figure legends.

Tables S1 mbio.00532-24-s0003.docx Sequences of primers used in this study.

Tables S2 mbio.00532-24-s0004.docx Sequences of siRNAs used in this study.

ASM does not own the copyrights to Supplemental Material that may be linked to, or accessed through, an article. The authors have granted ASM a non-exclusive, world-wide license to publish the Supplemental Material files. Please contact the corresponding author directly for reuse.
