# Supplementary figures and images for: PRRSV GP5 inhibits the antivirus effects of chaperone-mediated autophagy by targeting LAMP2A
Source: mBio. 2024 Jun 28;15(8):e00532-24. doi: 10.1128/mbio.00532-24 (PMC11323736; doi:10.1128/mbio.00532-24)

Figure S1

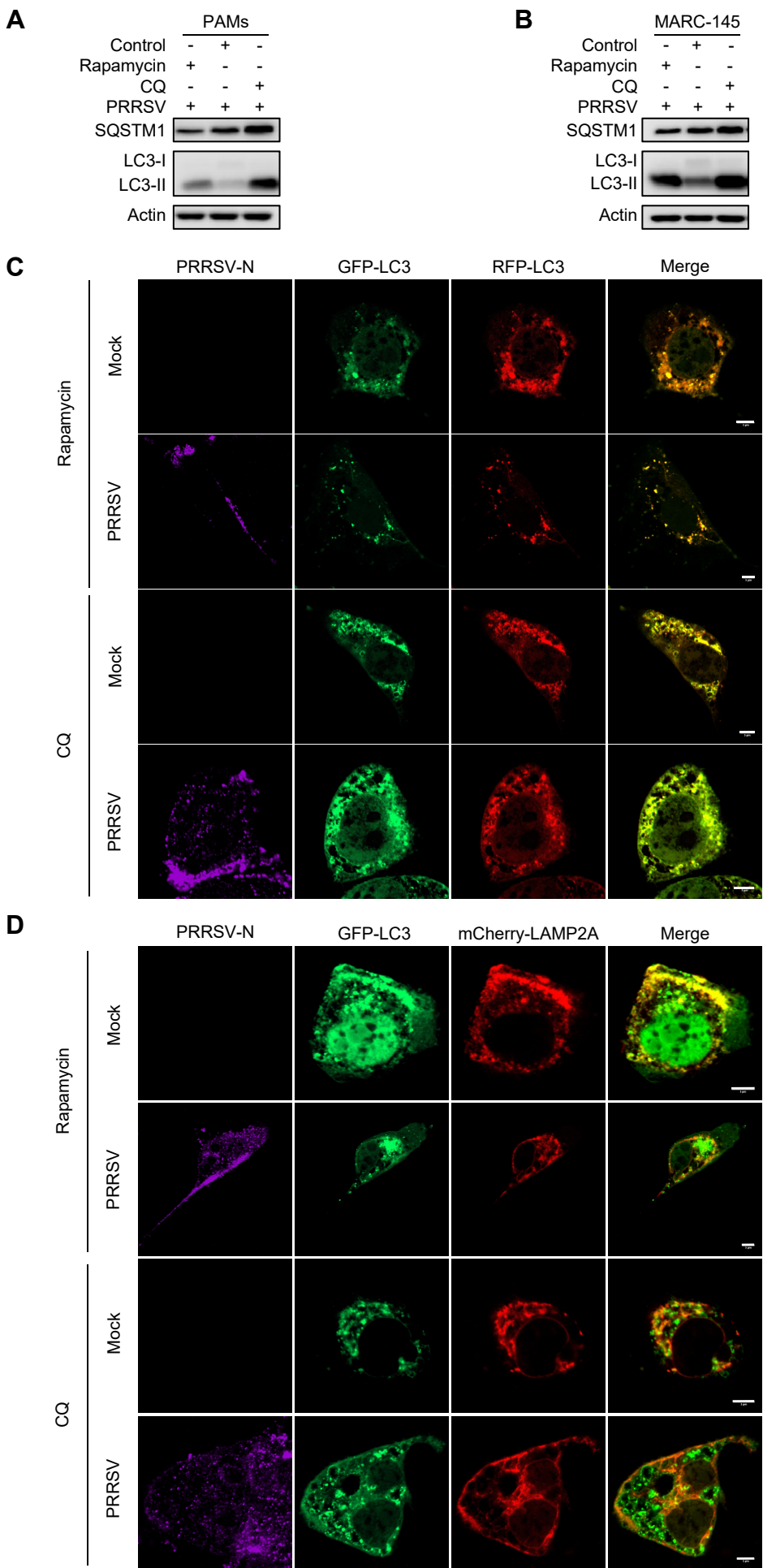

# Figure S2

**A**

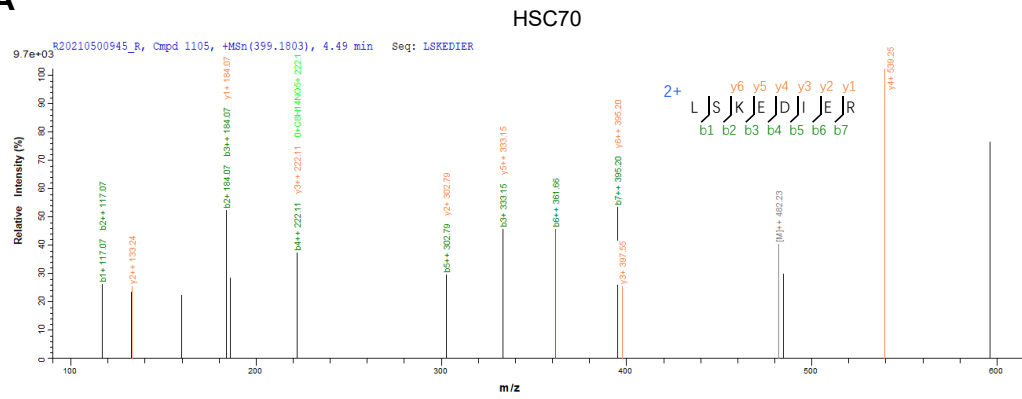

**B**

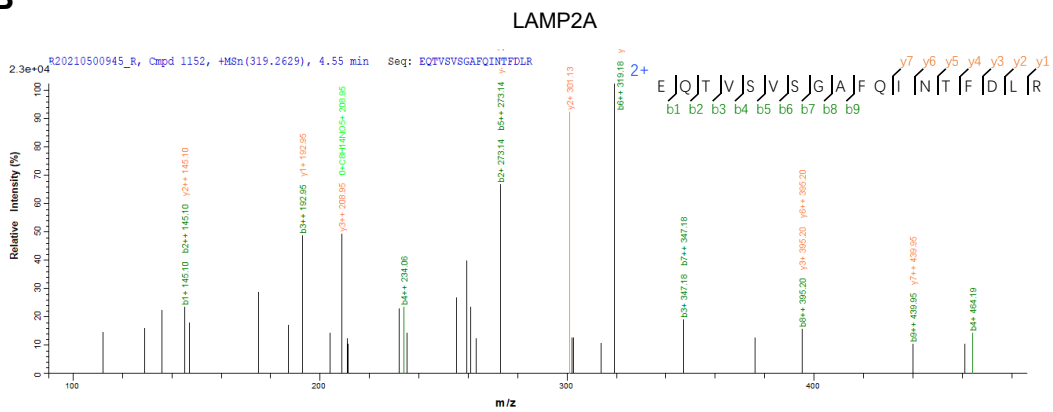

**C**

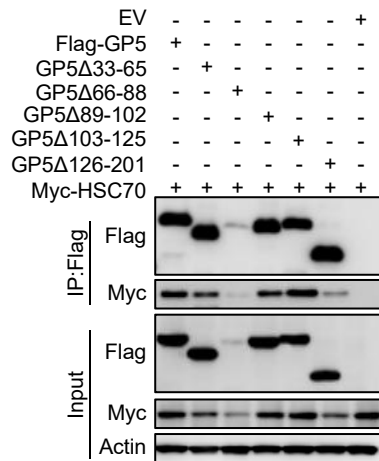

**D**

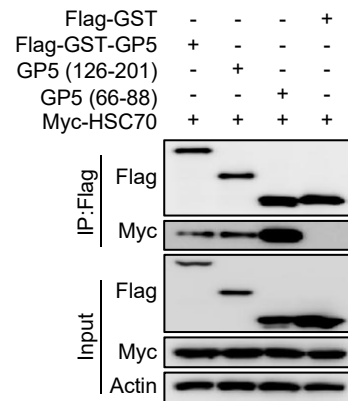

**E**

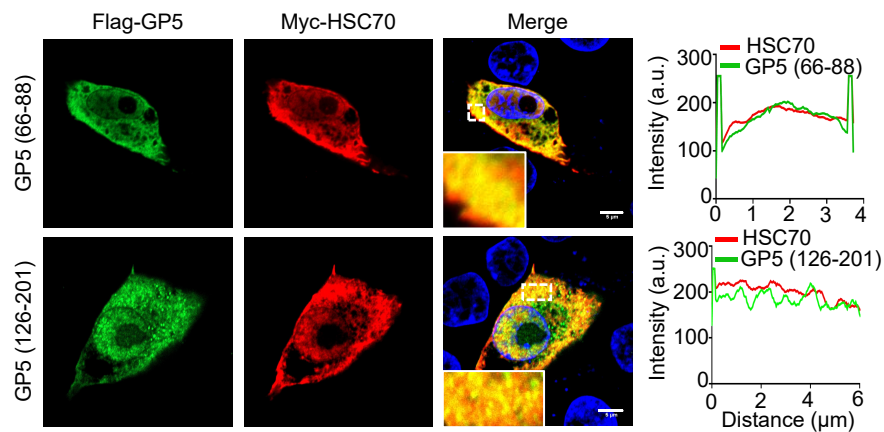

Figure S3

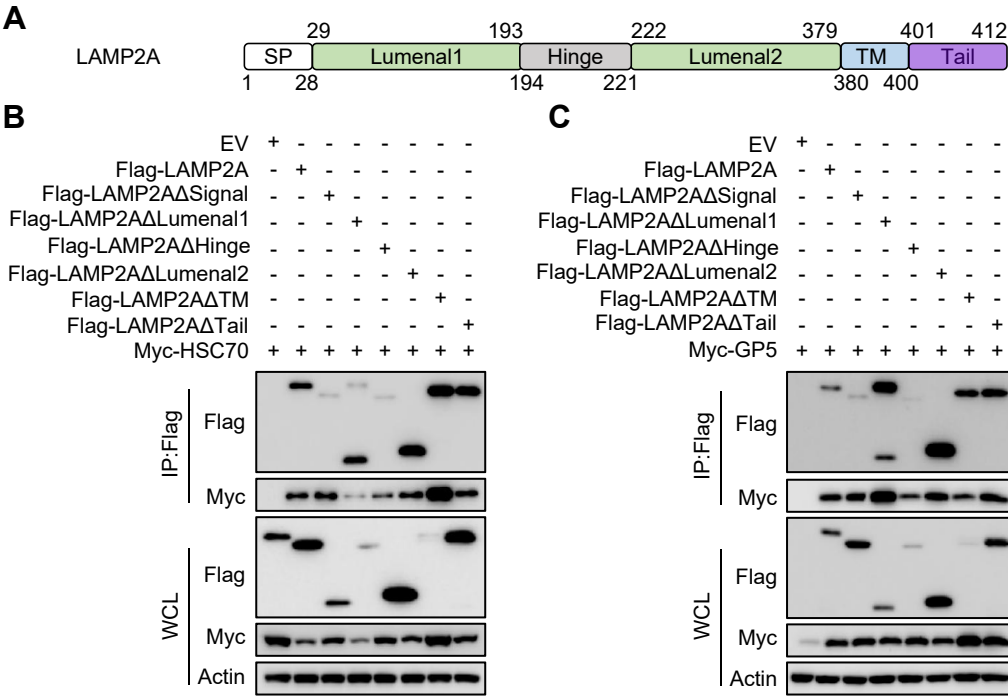

Figure S4

**A**

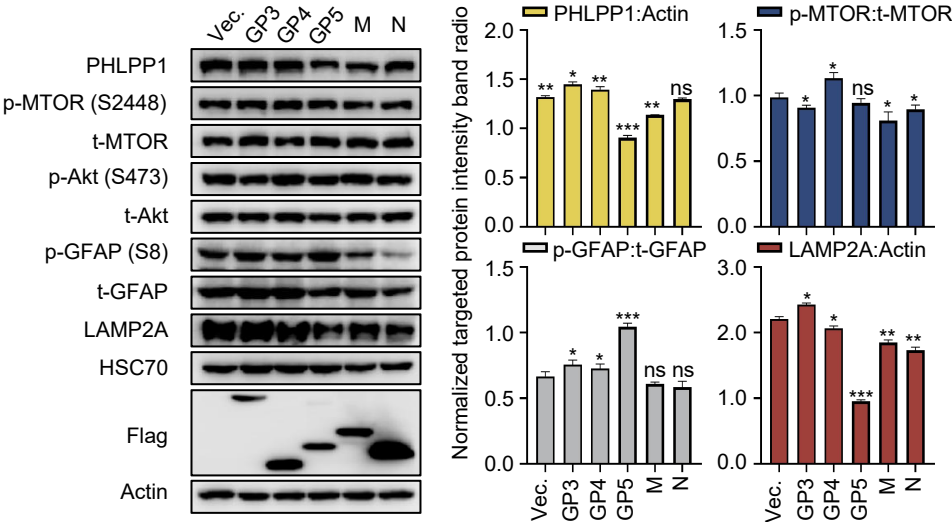

**B**

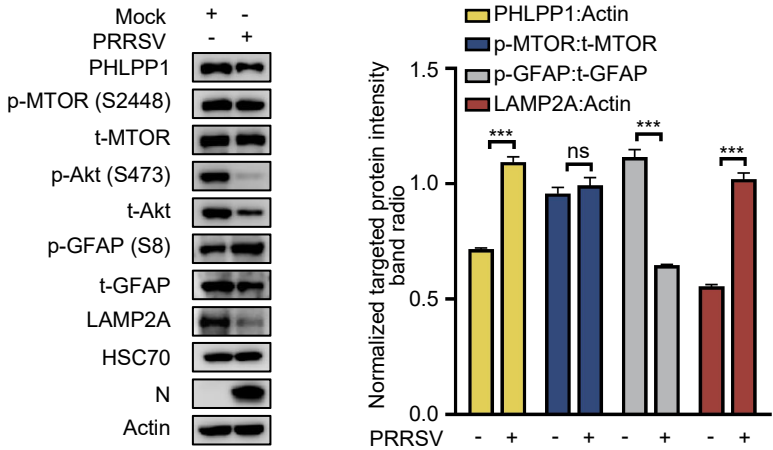

Figure S5

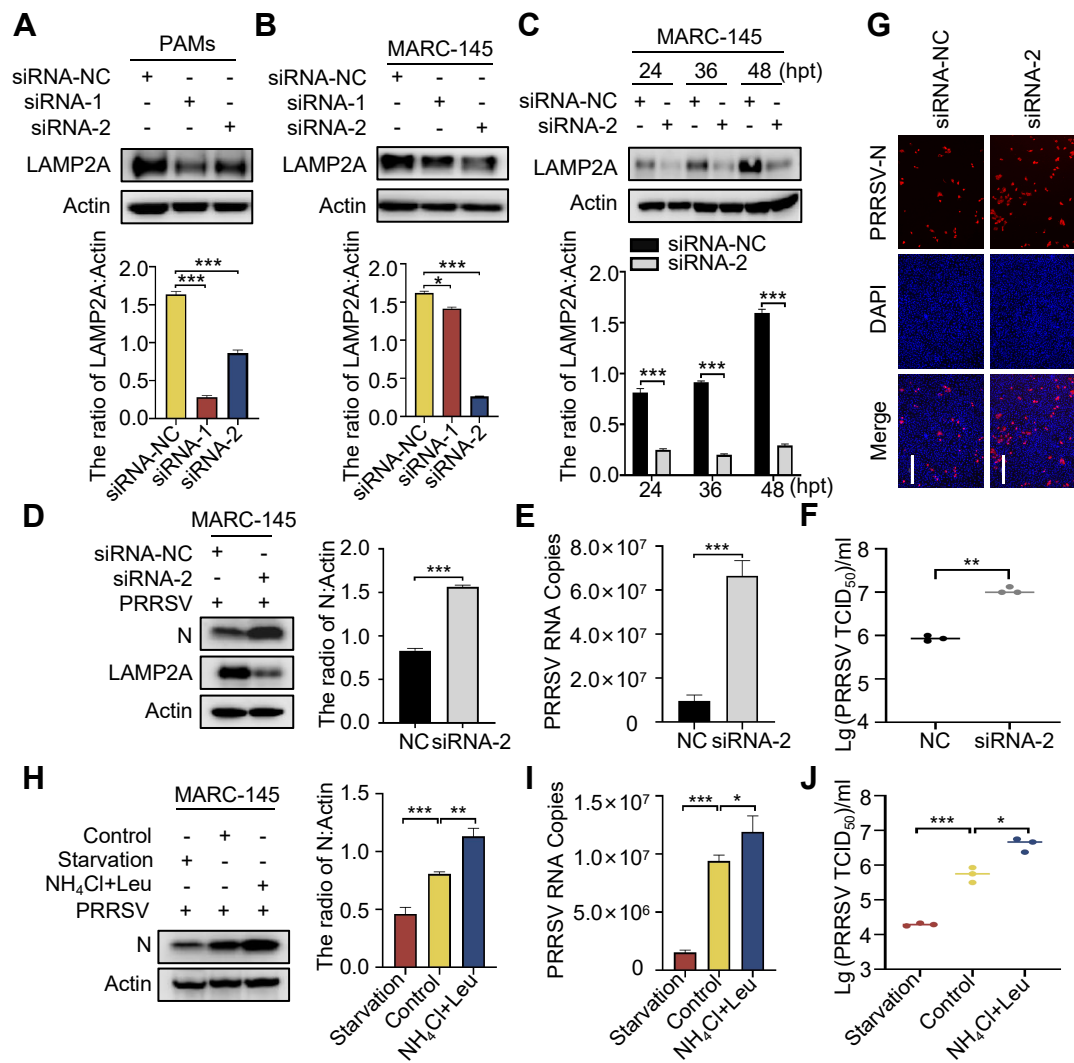

Figure S6

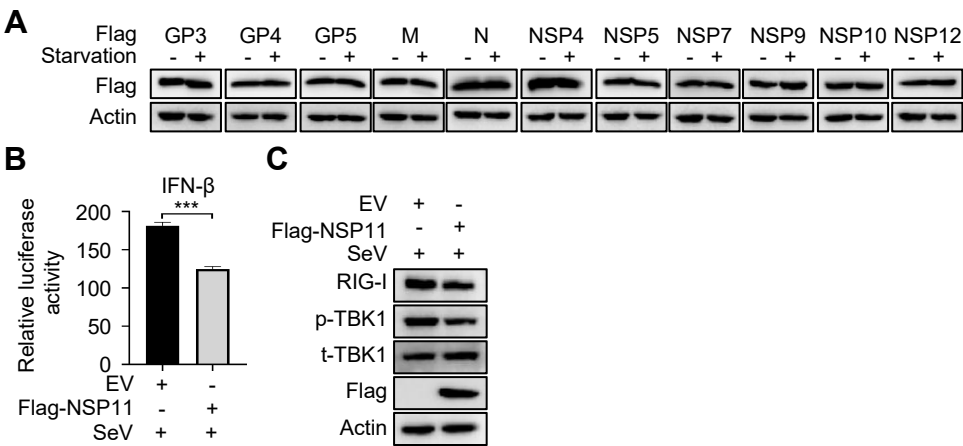

Supplement: Supplemental figures — Figures S1-S6. [file mbio.00532-24-s0001.pdf]
