# Supplementary material for: PRRSV GP5 inhibits the antivirus effects of chaperone-mediated autophagy by targeting LAMP2A
Source: mBio. 2024 Jun 28;15(8):e00532-24. doi: 10.1128/mbio.00532-24 (PMC11323736; doi:10.1128/mbio.00532-24)
Supplement: Legends — Supplemental figure legends. [file mbio.00532-24-s0002.docx]

**Supplemental figure legends**

**FIG S1** PRRSV infection inhibits the fusion between autophagosomes and lysosomes. (A, B) PAMs (A) or MARC-145 cells (B) were infected with PRRSV at an MOI of 1 for 8 h. Cells were treated with rapamycin (5 μM) or CQ (50 μM) for 6 h before collection. The resultant cell lysates were subjected to immunoblotting using the indicated antibodies. (C, D) MARC-145 cells, which were pre-transfected with RFP-GFP-LC3 (C), or GFP-LC3 and mCherry-LAMP2A (D) for 24 h, were infected with PRRSV at an MOI of 1 for 8 h. Cells were treated with rapamycin (5 μM) or CQ (50 μM) for 6 h before collection. The cells were immunostained with anti-PRRSV-N antibody, and observed under a confocal microscope. Scale bar indicates 5 μm.

**FIG S2** Identification of interaction domains between GP5 and HSC70. (A) LC‒MS/MS identification of HSC70. (B) LC‒MS/MS identification of LAMP2A. (C, D) HEK293T cells were cotransfected with the vectors expressing GP5 domain-deleted mutants and Myc-HSC70 (C), or vectors expressing GP5 domain-truncated mutants and Myc-HSC70 (D) for 24 h. The cell lysates were subjected to analysis of Flag-precipitation and immunoblotting analysis. 33-66 and 126-201: intracellular region, 66-88 and 103-125: transmembrane region, 89-102: extracellular region. (E) 3D4/21 cells were cotransfected with the vectors expressing GP5 domain-truncated mutants and Myc-HSC70. Cells were fixed and observed under a confocal microscope. Scale bar indicates 5 μm.

**FIG S3** Identification of interaction domains between LAMP2A and GP5. (A) Schematic representation of LAMP2A domains. SP: signal peptide, Lumenal 1: N-terminal first lumenal domain, Lumenal 2: C-terminal second lumenal domain, Tail: C-terminal cytosolic tail. (B, C) HEK293T cells were cotransfected with vectors expressing LAMP2A domain-deleted mutants and Myc-HSC70 (B), or vectors expressing LAMP2A domain-deleted mutants and Myc-GP5 (C) for 24 h. The cell lysates were subjected to analysis of Flag-precipitation and immunoblotting analysis.

**FIG S4** The effect of GP5 on CMA activity. (A) MARC-145 cells were transfected with vectors respectively expressing GP3, GP4, GP5, M, or N for 24 h. The resultant cell lysates were subjected to immunoblotting using the indicated antibodies. (B) MARC-145 cells were infected with PRRSV for 36 h. Cells were then subjected to immunoblotting using the indicated antibodies. Error bars: mean ± SD of 3 independent tests. Student's *t*-test; ns: non-significance; *p < 0.05; **p < 0.01; ***p < 0.001 compared to control.

**FIG S5** The effect of LAMP2A knockdown on PRRSV replication. (A, B) PAMs (A) or MARC-145 cells (B) were transfected with siRNA-1 and siRNA-2 against LAMP2A, or negative control siRNA-NC for 48 h. The knockdown efficiency was measured by western blotting. (C) MARC-145 cells were transfected with siRNA-2 against LAMP2A, or negative control siRNA-NC for the indicated times. The knockdown efficiency was measured by western blotting. (D-G) MARC-145 cells, which were pre-transfected with siRNA-LAMP2A, were infected with PRRSV for 36 h. Cells were harvested for western blotting (D), RT-qPCR (E), and IFA (G) analysis, and culture supernatants were collected for TCID_50_ measurement (F). (H-J) MARC-145 cells were infected with PRRSV for 24 h, and then treated with starvation for 16 h or treated with NH_4_Cl (10 mM) and leupeptin (Leu; 50 μM) for 12 h. Cells were harvested for western blotting (H) and RT-qPCR (I) analysis, and supernatants were collected for TCID_50_ measurement (J). Scale bar indicates 300 μm. Error bars: mean ± SD of 3 independent tests. Student's *t*-test; *p < 0.05; **p < 0.01; ***p < 0.001 compared to control.

**FIG S6** Activation of CMA decreases the expression of NSP11. (A) HEK293T cells were transfected with vectors expressing PRRSV viral proteins for 24 h, and then treated with starvation for 16 h. The cell lysates were subjected to immunoblotting analysis. (B) HEK293T cells were cotransfected with pIFN-β-luc, pRL-TK, and Flag-NSP11. After 24 h, the cells were infected with SeV (50 HAU) for 12 h, and then were analyzed by luciferase reporter assay system. (C) HEK293T cells were transfected with Flag-NSP11 for 24 h, and then infected with SeV (50 HAU) for 12 h before collection. The cell lysates were subjected to immunoblotting analysis. Error bars: mean ± SD of 3 independent tests. Student's *t*-test; ***p < 0.001 compared to control.
