# Supplementary material for: PRRSV GP5 inhibits the antivirus effects of chaperone-mediated autophagy by targeting LAMP2A
Source: mBio. 2024 Jun 28;15(8):e00532-24. doi: 10.1128/mbio.00532-24 (PMC11323736; doi:10.1128/mbio.00532-24)
Supplement: Tables S1 — Sequences of primers used in this study. [file mbio.00532-24-s0003.docx]

**Table S1**. The sequences of primers used in this study.

| Name^a^ | Sequences (5ʹ to 3ʹ)^b^ | Product size |
| --- | --- | --- |
| GFP-GP5-F | CCG*CTCGAG*AACAACAGCAGCTCTCATATTCAGT | 510bp |
| GFP-GP5-R | CCG*GAATTC*CTATAGACGACCCCATCGTTCC |  |
| Flag-GP5-F | CCC*AAGCTT*AGCAACAACAGCAGCTCTCATAT | 510bp |
| Flag-GP5-R | CCG*CTCGAG*CTATAGACGACCCCATCGTTCC |  |
| Flag-GP5ΔCMA-1F | CCC*AAGCTT*AGCAACAACAGCAGCTCTC | 87bp |
| Flag-GP5ΔCMA-1R | CTCCACTGCTGTCAGCCAATCTGTGCC |  |
| Flag-GP5ΔCMA-1’F | TGGCTGACAGCAGTGGAGACTTTTGTCAT | 426bp |
| Flag-GP5ΔCMA-1’R | CCG*CTCGAG*CTATAGACGACCCCATCGT |  |
| Flag-GP5 (QK58-59AA)-1F | CCG*GAATTC*GGAGCAACAACAGCAGCTCTCAT | 87bp |
| Flag-GP5 (QK58-59AA)-1R | AAATGCTGCTGTCAGCCAATCTGTGCCAT |  |
| Flag-GP5 (QK58-59AA)-1’F | ctgacaGCAGCATTTGACTGGGCAGTGGAGACT | 438bp |
| Flag-GP5 (QK58-59AA)-1’R | CCG*CTCGAG*CTATAGACGACCCCATCGTTCC |  |
| FlagGST-GP5-F | ccg*GAATTC*GGAACAACAGCAGCTCTCATATTCAGT | 507bp |
| FlagGST-GP5-R | ccg*CTCGAG*CTATAGACGACCCCATCGTTCC |  |
| FlagGST-GP5Δ33-65-F | CCG*GAATTC*GGACTTTTGTCATCTTCCCCGTGTT | 408bp |
| FlagGST-GP5Δ33-65-R | CCG*CTCGAG*CTATAGACGACCCCATCGTTCC |  |
| FlagGST-GP5Δ66-88-1F | CCG*GAATTC*GGAACAACAGCAGCTCTCATATTCAGT | 114bp |
| FlagGST-GP5Δ66-88-1R | TCGCTGCCGCCACCGCCCTCCACTGCCCAGTCAAA |  |
| FlagGST-GP5Δ66-88-1’F | AGGGCGGTGGCGGCAGCGACACAGTTGGTCTGGCC | 354bp |
| FlagGST-GP5Δ66-88-1’R | CCG*CTCGAG*CTATAGACGACCCCATCGTTCC |  |
| FlagGST-GP5Δ89-102-1F | CCG*GAATTC*GGAACAACAGCAGCTCTCATATTCAGT | 183bp |
| FlagGST-GP5Δ89-102-1R | GTGGCTGCCGCCACCGCCAAGGAAATGGCTGGTGG |  |
| FlagGST-GP5Δ89-102-1’F | CTTGGCGGTGGCGGCAGCCACGGGCGGTATGTCTT | 312bp |
| FlagGST-GP5Δ89-102-1’R | CCG*CTCGAG*CTATAGACGACCCCATCGTTCC |  |
| FlagGST-GP5Δ103-125-1F | CCG*GAATTC*GGAACAACAGCAGCTCTCATATTCAGT | 225bp |
| FlagGST-GP5Δ103-125-1R | TGCTGCCGCCACCGCCATAATATCCGGCGGTGGAC |  |
| FlagGST-GP5Δ103-125-1’F | ATGGCGGTGGCGGCAGCAGGCTTGCGAAGAACTGC | 243bp |
| FlagGST-GP5Δ103-125-1’R | CCG*CTCGAG*CTATAGACGACCCCATCGTTCC |  |
| FlagGST-GP5Δ126-201-F | CCG*GAATTC*GGAACAACAGCAGCTCTCATATTCAGT | 282bp |
| FlagGST-GP5Δ126-201-R | CCG*CTCGAG*CTAAATGACAAAGCAAATCAACGC |  |
| FlagGST-GP5 (66-88)-F | ccg*GAATTC*ggACTTTTGTCATCTTCCCCGT | 72bp |
| FlagGST-GP5 (66-88)-R | ccg*CTCGAG*aTTAAAGGAAATGGCTGGTGGTGAG |  |
| FlagGST-GP5 (126-201)-F | ccg*GAATTC*ggAGGCTTGCGAAGAACTGCAT | 228bp |
| FlagGST-GP5 (126-201)-R | ccg*CTCGAG*aCTATAGACGACCCCATCGTTCC |  |
| GFP-LC3-F | CCC*AAGCTT*ATGCCCTCAGACCGGCCT | 366bp |
| GFP-LC3-R | CGC*GGATCC*TCAGAAGCCGAAGGTTTCC |  |
| Myc-HSC70-F | CCG*CTCGAG*GTATGTCTAAGGGACCTGCAGT | 1941bp |
| Myc-HSC70-R | ATTT*GCGGCCGC*TTAGTCAACCTCCTCAATGGT |  |
| Flag-LAMP2A-F | CCG*GAATTC*GGATGGTGTGCTTCCGCCT | 1236bp |
| Flag-LAMP2A-R | CCG*CTCGAG*CTAAAATTGCTCATATCCAGC |  |
| mCherry-LAMP2A-F | CCG*CTCGAG*CTATGGTGTGCTTCCGCCT | 1236bp |
| mCherry-LAMP2A-R | CCG*GAATTC*CTAAAATTGCTCATATCCAGC |  |
| Flag-LAMP2AΔSignal-F | ccg*GAATTC*ggTTGGAACTTAATTTGACAAATTCA | 1152bp |
| Flag-LAMP2AΔSignal-R | ccg*CTCGAG*CTAAAATTGCTCATATCCAGCATG |  |
| Flag-LAMP2AΔLumenal1-1F | ccg*GAATTC*ggATGGTGTGCTTCCGCCT | 92bp |
| Flag-LAMP2AΔLumenal1-1R | gttaaagtTGCATAAGACGAGACAGCTCC |  |

**Continued Table S1**. The sequences of primers used in this study.

| Name^a^ | Sequences (5ʹ to 3ʹ)^b^ | Product size |
| --- | --- | --- |
| Flag-LAMP2AΔLumenal1-1’F | cttatgcaACTTTAACTACAACGGTGCCC | 665bp |
| Flag-LAMP2AΔLumenal1-1’R | ccg*CTCGAG*CTAAAATTGCTCATATCCAGCATG |  |
| Flag-LAMP2AΔHinge-1F | ccg*GAATTC*ggATGGTGTGCTTCCGCCT | 587bp |
| Flag-LAMP2AΔHinge-1R | TAGGATCCCTTATCTTTATCACACAAAAACTCT |  |
| Flag-LAMP2AΔHinge-1’F | aagataagGGATCCTATTCAGTTACAAGTAGCA | 581bp |
| Flag-LAMP2AΔHinge-1’R | ccg*CTCGAG*CTAAAATTGCTCATATCCAGCATG |  |
| Flag-LAMP2AΔLumenal2-1F | ccg*GAATTC*ggATGGTGTGCTTCCGCCT | 671bp |
| Flag-LAMP2AΔLumenal2-1R | ACCGCTATAGTCTCCGGTTTTTCCTTTG |  |
| Flag-LAMP2AΔLumenal2-1’F | cggagactATAGCGGTGGGAGCAGCC | 107bp |
| Flag-LAMP2AΔLumenal2-1’R | ccg*CTCGAG*CTAAAATTGCTCATATCCAGCATG |  |
| Flag-LAMP2AΔTM-1F | ccg*GAATTC*ggATGGTGTGCTTCCGCCT | 1145bp |
| Flag-LAMP2AΔTM-1R | CGCTTGAGGGGCACAATGAAGTTGTCGTCA |  |
| Flag-LAMP2AΔTM-1’F | ttgtgcccCTCAAGCGCCATCATGCTG | 44bp |
| Flag-LAMP2AΔTM-1’R | ccg*CTCGAG*CTAAAATTGCTCATATCCAGCATG |  |
| Flag-LAMP2AΔTail-F | ccg*GAATTC*ggATGGTGTGCTTCCGCCTCTC | 1203bp |
| Flag-LAMP2AΔTail-R | ccg*CTCGAG*aCTAGAGACCAATAAAGTAAGCCAGC |  |
| PAmCherry-KFERQ-F | cta*gctagc*gccaccATGAAGGAAACTGCAGCAGCCAAGTTTGAGCGGCAGCA | 87bp |
| PAmCherry-KFERQ-R | cgc*ggatcc*CGCAGCGGAAGTGCTGGAGTCCATGTGCTGCCGCTCAAAC |  |
| Flag-EF1α-F | acgc*GTCGAC*CATGGGAAAGGAGAAGACTCACATC | 1389bp |
| Flag-EF1α-R | cgg*GGTACC*TCATTTAGCCTTCTGAGCTTTCTG |  |
| Flag/GFP-GFAP-F | CCC*AAGCTT*ATGGAGAGGAGACGGGTCAC | 1407bp |
| Flag/GFP-GFAP-R | CCG*GAATTC*TCACACCACATCCTTGTGCT |  |
| Flag-GFAP (S8D)-F | atggagaggagaCGggtcaccGATgcggctcgc | 5186bp |
| Flag-GFAP (S8D)-R | gacataggagcggcgagccgcATCggtgacccg |  |
| GFP-GFAP (S8D)-F | atggagaggagaCGggtcaccGATgcggctcgc | 6107bp |
| GFP-GFAP (S8D)-R | gacataggagcggcgagccgcATCggtgacccg |  |
| GFP-GFAP (S8A)-F | atggagaggagacgggtcaccGcagcggctcgc | 6107bp |
| GFP-GFAP (S8A)-R | gacataggagcggcgagccgctgCggtgacccg |  |
| LAMP2A-qF | GTTCTGGTCTGCCTCGTC | 176bp |
| LAMP2A-qF | GTCACAGTGCCACGGTCT |  |
| PRRSV-ORF7-qF | AAACCAGTCCAGAGGCAAGG | 221bp |
| PRRSV-ORF7-qR | GCAAACTAAACTCCACAGTGTAA |  |
| β-Actin-qF | GTGACGTGGACATCCGTAAA | 107bp |
| β-Actin-qR | CAGGGCAGTAATCTCCTTCTG |  |

^a^ F: forward primer, R: reverse primer.

^b^ The italicized alphabets indicate restriction enzyme cleavage sites for cloning.

The “q” in the Name represents the primers used for RT-qPCR.
