# Supplementary material for: PRRSV GP5 inhibits the antivirus effects of chaperone-mediated autophagy by targeting LAMP2A
Source: mBio. 2024 Jun 28;15(8):e00532-24. doi: 10.1128/mbio.00532-24 (PMC11323736; doi:10.1128/mbio.00532-24)
Supplement: Tables S2 — Sequences of siRNAs used in this study. [file mbio.00532-24-s0004.docx]

**Table S2**. The sequences of siRNAs used in this study.

| Name | Forward sequence (5ʹ-3ʹ) | Reverse sequence (5ʹ-3ʹ) |
| --- | --- | --- |
| siRNA-1 | GGAGACUGGAUCCUAUUCATT | UGAAUAGGAUCCAGUCUCCTT |
| siRNA-2 | GGCAGAUGAAUUUCACAAUTT | AUUGUGAAAUUCAUCUGCCTT |
| siRNA-NC | UUCUCCGAACGUGUCACGUTT | ACGUGACACGUUCGGAGAATT |
